# USP12 facilitates gastric cancer progression via stabilizing YAP

**DOI:** 10.1038/s41420-024-01943-2

**Published:** 2024-04-11

**Authors:** Peng Zhang, Dongyi Liu, Yifeng Zang, Jinqing Wang, Ziping Liu, Jian Zhu, Xin Li, Yinlu Ding

**Affiliations:** 1 Department of General Surgery, The Second Hospital, Cheeloo College of Medicine, Shandong University, Jinan, 250033 Shandong PR China; 2 Department of Anaesthesiology, The Second Hospital, Cheeloo College of Medicine, Shandong University, Jinan, 250033 Shandong PR China; 3 Department of General Surgery, Shengjing Hospital of China Medical University, Shenyang, 110000 Liaoning PR China; 4 Xinxiang Key Laboratory of Tumor Migration and Invasion Precision Medicine, School of Medical Technology, Xinxiang Medical University, Xinxiang, 453003 Henan PR China; 5 Department of Surgical Oncology and General Surgery, The First Hospital of China Medical University, Shenyang, 110000 Liaoning PR China

**Keywords:** Gastric cancer, Oncogenes

## Abstract

The dysregulation of Hippo signaling is a crucial factor driving the progression of gastric cancer, making the targeting of the Hippo pathway a promising therapeutic strategy. However, effective drugs targeting the Hippo/YAP axis remain unavailable. Thus, identifying potential therapeutic targets and mechanisms that inhibit the activity of the Hippo/YAP axis in gastric cancer is of paramount importance. The ubiquitination modification of the Hippo/YAP pathway plays a significant role in signaling transduction and cancer progression. In an effort to shed light on effective therapeutic targets, we conducted a screening using a deubiquitinase small interfering RNA library, leading to the identification of USP12 as an important deubiquitinase in the context of Hippo/YAP axis and the progression of gastric cancer. Our bioinformatic analysis further demonstrated a correlation between USP12 and poor survival, as well as a positive association with classical YAP target genes in gastric cancer samples. Notably, USP12 depletion was found to inhibit gastric cancer progression via the Hippo/YAP axis, whereas USP12 overexpression exhibited the opposite effect, promoting gastric cancer growth and enhancing YAP activity. Further studies through immuno-staining and immuno-precipitation assays indicated the nuclear localization of USP12 and its association with YAP to enhance YAP stability. Specifically, our findings revealed that USP12 could inhibit K48-linked poly-ubiquitination of YAP, predominantly at the K315 site. As a result, we have identified a novel regulatory mechanism involving USP12 and Hippo signaling in the progression of gastric cancer, with the potential for blockade of USP12 to materialize as a promising strategy for combating gastric cancer.

## Introduction

Gastric cancer is a prevalent malignancy of the digestive system. According to the recent cancer statistics, gastric cancer incidence and mortality in China rank NO.3 [1, 2]. Despite the potential for surgical treatment, the 5-year survival rate remains at 30%, presenting an urgent clinical challenge [3]. With the introduction of personalized target therapy, several genome-wide association studies aimed to identify the molecular classifications of gastric cancer, which included Epstein-Barr virus, chromosomal instability, microsatellite instability, and genomic stable subtype. However, these molecular classifications have provided limited guidance for clinical therapy [4]. Additionally, while several immunotherapy and target therapy drugs have been successfully used in treating gastric cancer, the therapeutic options are still limited [5]. Thus, identifying new therapeutic targets for treating gastric cancer is a pressing and crucial task.

The Hippo signaling pathway was initially identified in Drosophila, and further studies have shown its important functions in maintaining physiological balance and contributing to human cancers [6]. The Hippo pathway consists of a series of phosphorylation mediated by a set of phospho-kinases. Activation of the Hippo signaling pathway leads to phosphorylation of LATS1/2 by the kinase MST1/2. Subsequently, YAP is phosphorylated by phosphorylated LATS1/2 at multiple serine/threonine sites, which causes its retention in the cytoplasm. The phosphorylated YAP protein then interacts with β-TrCP, ultimately resulting in the degradation of YAP through the proteasome pathway [7]. In the absence of Hippo pathway activity, unphosphorylated YAP protein translocates to the nucleus and interacts with various transcription factors like TEADs to enhance the expression of target genes and drive tumorigenesis [8]. Moreover, YAP can function as a co-repressors of target genes, facilitating tumor suppression and pro-apoptotic cell death in response to DNA damage or other pro-apoptotic signals [9].

In recent years, Hippo signaling affects the progression of gastric cancer has been demonstrated [10, 11]. Gastric cancer often shows abnormalities in the Hippo signaling pathway, with higher levels of YAP expression detected in gastric cancer samples than in normal gastric tissues. In gastric cancers, YAP expression, lymph node metastasis, and poor survival were found to be correlated based on survival analysis [12]. The biological data shows that YAP is crucial for the progression of gastric cancer in both in vivo and in vitro settings. Additionally, blocking the YAP-TEAD interaction was enough to inhibit the advancement of gastric cancer [13, 14]. Interestingly, recent studies revealed that YAP was involved in the transformation of gastritis to gastric cancer, possibility via trans-activation of Wnt pathway [15]. Based on the important function of Hippo signaling, we propose that target YAP protein may be a possible strategy for the treatment of gastric cancer.

Despite the tight control of the Hippo pathway activation through the MST/LATS/YAP phosphorylation cascade, the development of effective modulators targeting the cancer therapeutic pathway is still in its early stages [16]. Furthermore, the persistent over-activation of YAP in gastric cancer, despite the functional restrictive kinase cascade, presents a perplexing mystery. Recent findings have revealed the significance of ubiquitin modification in modulating YAP function and Hippo kinase activity [17]. Specifically, a group of E3 ubiquitin ligases and deubiquitinases (DUBs) delicately control the protein stability of YAP, with selective inhibition of certain DUBs having the potential to reverse this balance and facilitate YAP degradation [18–21]. Our study seeks to identify the critical deubiquitinase that targets YAP stability. The deubiquitinase siRNA library screen identified USP12 as a potential deubiquitinase involved in gastric cancer progression and the Hippo/YAP axis. USP12 was found to be upregulated in patients with gastric cancer and was associated with poor survival in gastric cancer. Furthermore, bioinformatic analysis revealed a possible association between USP12 and the YAP signature gene cluster in gastric cancer. Subsequent biological studies confirmed the essential role of USP12 in the Hippo/YAP axis and the progression of gastric cancer. In conclusion, our findings suggest that USP12 plays an intriguing regulatory role in the modulation of Hippo signaling in gastric cancer, suggesting that USP12 could serve as a potential target for the Hippo/YAP axis in the context of gastric cancer.

## Results

### USP12 plays a crucial role in regulating Hippo-YAP signaling in gastric cancer

**Fig. 1:**
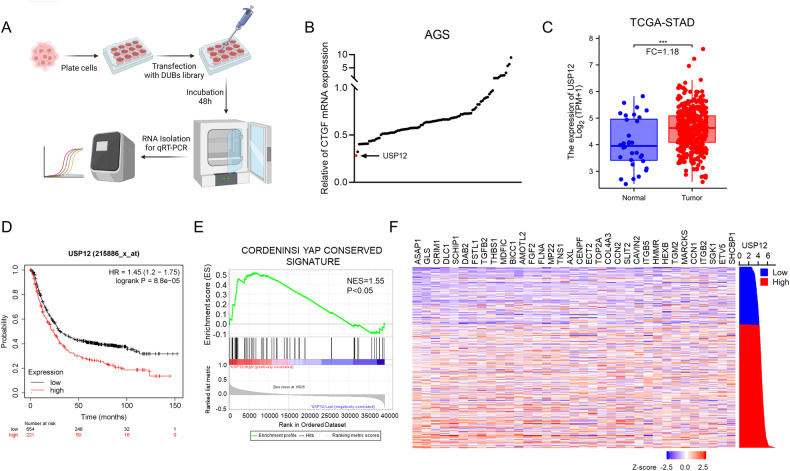
USP12 is highly expressed and linked to a poor prognosis, correlating with the Hippo/YAP signaling pathway in gastric cancer. **A** Flow chart of deubiquitinating enzyme library screening in AGS cell lines. Transfect AGS cells with 20uM siRNA to knockdown each of the 96 human DUB genes. After 48 h, quantitative analysis of gene expression is performed by qRT-PCR. **B** The classical target gene CTGF was used to indicate YAP. The siRNA screening data demonstrated that USP12 was required for CTGF gene expression in AGS cells. **C** The expression of USP12 is significantly increased in gastric cancer tissues compared to normal tissues in the TCGA database (FC = 1.18, *P* < 0.001). *P < 0.05; **P < 0.01; ***P < 0.001. **D** Kaplan−Meier analysis comparing the overall shows that high USP12 expression is associated with poor prognosis in gastric cancer patients from public meta-analysis data (https://kmplot.com/analysis/). **E** The TCGA database provided transcriptome samples from gastric cancer patients, which were divided into two cohorts based on USP12 expression levels (high vs. low, 50% each). Gene Set Enrichment Analysis (GSEA) was conducted to assess the impact of USP12 expression levels on the enrichment of YAP signature genes. **F** Heatmap of correlation analysis between USP12 expression and YAP target genes in TCGA data of gastric cancer.

To identify key DUBs that control Hippo signaling in gastric cancer, we screened an important siRNA library targeting DUBs (Fig. 1A). AGS cells are a commonly used cell line in gastric cancer research, and we used AGS cells as a screening model. Given CTGF is known as a classical target gene in the Hippo/YAP pathway, we utilized CTGF as an indicator of Hippo/YAP signaling activity. In AGS cells, knockdown of USP12 showed the most significant modulation of CTGF expression among the deubiquitin enzyme library. (Fig. 1B). We conducted additional research on USP12 expression in human gastric cancer, revealing elevated levels of USP12 in gastric cancer according to data from the TCGA database (*p* < 0.001, FC = 1.18, Fig. 1C). Besides, the KMplot database analysis revealed that USP12 is associated with poor survival in gastric cancer patients. (Fig. 1D). we explored the relationship between USP12 and the Hippo-YAP pathway using TCGA database information. To achieve this, we categorized the transcriptome samples of gastric cancer samples in the TCGA database into two cohorts based on high and low USP12 expression levels (50% vs. 50%). The GSEA conducted that YAP signature genes were enriched in the group with elevated USP12 expression (Fig. 1E). According to the TCGA database, USP12 correlated positively with several YAP target genes, including CCN1 (CYR61) and CCN2 (CTGF) (Fig. 1F). The results indicate that high USP12 expression is linked to a poor prognosis in gastric cancer and may be a crucial oncogene of the disease.

### USP12 is essential for the progression of gastric cancer

**Fig. 2:**
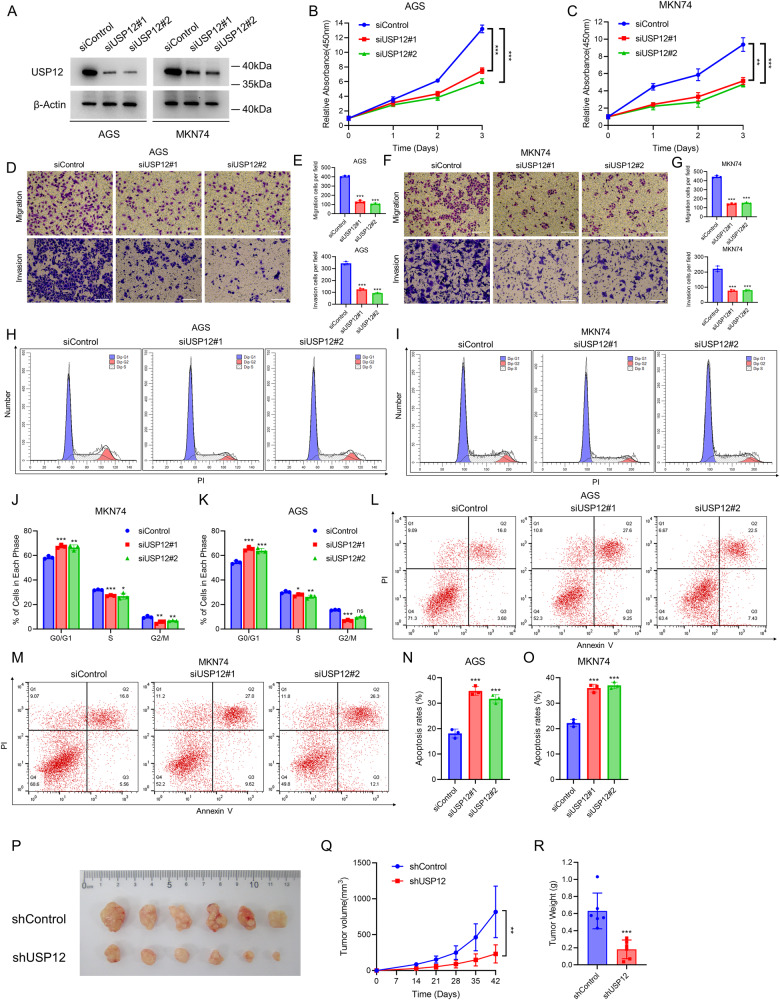
USP12 depletion inhibits cell proliferation and promotes cell apoptosis in gastric cancer cells. **A** Western blotting demonstrated knockdown efficiency of USP12. AGS and MKN74 cells transfected with 50 nM siControl or two independent siUSP12. β-Actin was used as the internal control. **B**, **C** USP12 depletion inhibits the proliferation of gastric cancer cells. AGS and MKN74 cells were transfected with 50 nM siControl or two independent siUSP12. After 24 h, CCK-8 assay was used to determine cell absorbance at the indicated time points after transfection. Experiments were performed in triplicate. *P < 0.05; **P < 0.01; ***P < 0.001 for cell growth comparisons. **D–G** USP12 depletion reduced the number of migration and invasion gastric cancer cells. AGS and MKN74 cells were transfected with 50 nM siControl or two independent siUSP12. After 24 h, siControl and siUSP12 cells were separately plated into the Transwell chambers. For invasion experiments, it is necessary to spread the matrix gel before planting the cells. After 12 h, the cells were fixed and stained with crystal violet. The transwell cell number was determined to indicate cell migration (upper) and invasion (lower) activity (**D**, **F**). Quantification of transwell results using ImageJ software (**E**, **G**). Scale bar 100 μm. *N* = 3, *P < 0.05; **P < 0.01; ***P < 0.001 for cell migration comparisons. **H–K** Cell-cycle analysis by flow cytometry of AGS and MKN74 cells transfected with 50 nM siControl or two independent siUSP12. After 24 h, the cells were harvested, fixed with 70% ethanol, and stained with propidium iodide. The cells were subjected to FACS analysis (**H**, **I**). Quantitative summary of cell-cycle analysis using graphpad software (**J**, **K**). Experiments were performed in triplicate. *P < 0.05; **P < 0.01; ***P < 0.001 for cell proportion comparisons. **L–O** Representative plots of apoptosis AGS and MKN74 cells transfected with 50 nM siControl or two independent siUSP12. After 24 h, the cells were harvested, and stained with Annexin V-PI (**L**, **M**). Quantitative summary of apoptosis analysis using graphpad software (**N**, **O**). Experiments were performed in triplicate. *P < 0.05; **P < 0.01; ***P < 0.001 for cell proportion comparisons. **P**–**R** USP12 depletion inhibits the tumor growth of AGS cells in the xenograft model. Harvested and photographed tumors in the shUSP12 and shControl (**P**), tumor volume (**Q**) and weight (**R**) growth in each mouse from the shUSP12 and the shControl in vivo. *P < 0.05; **P < 0.01; ***P < 0.001.

Due to USP12 as an oncogene in gastric cancer, we further explored the biological function in gastric cancer cell lines. For this research, the AGS and MKN74 cell lines were selected as cell models for this study. We used western blot assay to confirm the efficient knockdown of USP12 (Fig. 2A). CCK8 assays showed that deletion of USP12 inhibited gastric cancer cell proliferation (Fig. 2B, C). Additionally, the transwell assay revealed that USP12 deletion decreased cell invasion and migration (Fig. 2D–G). Furthermore, PI staining by flow cytometry analysis revealed that USP12 deletion significantly increased G0/G1 phase cell proliferation (Fig. 2H–K). Annexin V-PI apoptosis staining demonstrated that deletion of USP12 resulted in an increased of apoptotic cells (Fig. 2L–O). Moreover, our results were validated in xenograft model demonstrating that the depletion of USP12 significantly hindered the growth of gastric cancer tumors in living organisms (Fig. 2P–R). The in vivo and in vitro experiments demonstrated that USP12 is necessary for the growth of gastric cancer.

### The progression of gastric cancer is regulated by USP12 through its deubiquitination function

**Fig. 3:**
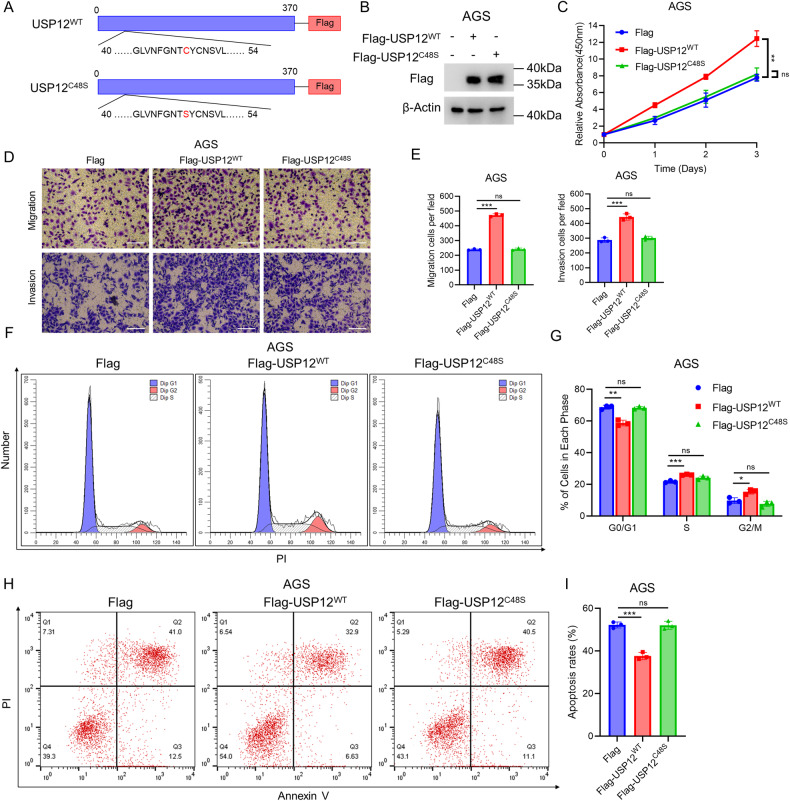
USP12^WT^ but not USP12^C48S^ promotes cell proliferation and inhibits cell apoptosis in gastric cancer cells. **A** Schematic structure of the USP12 wild-type (Flag-USP12^WT^) and enzyme inactivation mutant (Flag-USP12^C48S^). **B** Western blotting demonstrated overexpression of USP12^WT^ and USP12^C48S^. AGS cells transfected with 1 μg Flag or Flag-USP12^WT^/Flag-USP12^C48S^. β-Actin was used as the internal control. **C** USP12^WT^ but not USP12^C48S^ promotes cell proliferation. AGS cells were transfected with 1 μg Flag or Flag-USP12^WT^/Flag-USP12^C48S^. After 24 h, CCK-8 assay was used to determine cell absorbance at the indicated time points after transfection. Experiments were performed in triplicate. *P < 0.05; **P < 0.01; ***P < 0.001 for cell growth comparisons. **D**, **E** USP12^WT^ but not USP12^C48S^ promotes cell migration. AGS cells were transfected with 1 μg Flag or Flag-USP12^WT^/Flag-USP12^C48S^. After 24 h, cells were separately plated into the Transwell chambers. For invasion experiments, it is necessary to spread the matrix gel before planting the cells. After 12 h, the cells were fixed and stained with crystal violet. The transwell cell number was determined to indicate cell migration (upper) and invasion (lower) activity (**D**). Quantification of transwell results using ImageJ software (**E**). Scale bar 100 μm. *N* = 3, *P < 0.05; **P < 0.01; ***P < 0.001 for cell migration comparisons. **F**, **G** USP12^WT^ but not USP12^C48S^ promotes cell cycle progression. AGS cells were transfected with 1 μg Flag or Flag-USP12^WT^/Flag-USP12^C48S^. After 24 h, the cells were harvested, fixed with 70% ethanol, and stained with propidium iodide. The cells were subjected to FACS analysis (**F**). Quantitative summary of cell-cycle analysis using graphpad software (**G**). Experiments were performed in triplicate. *P < 0.05; **P < 0.01; ***P < 0.001 for cell proportion comparisons. **H**, **I** Representative plots of apoptosis AGS cells transfected with 1 μg Flag or Flag-USP12^WT^/Flag-USP12^C48S^. After 24 h, the cells were harvested, and stained with Annexin V-PI (**H**). Quantitative summary of apoptosis analysis using graphpad software (**I**). Experiments were performed in triplicate. *P < 0.05; **P < 0.01; ***P < 0.001 for cell proportion comparisons.

To assess the catalytic activity of deubiquitinating enzymes in USP12 contributes to the phenotype of gastric cancer cells, we overexpressed USP12 wild-type (USP12^WT^) and USP12 enzyme-inactivated mutants [22] (USP12^C48S^, Fig. 3A, B). Elevated levels of cell proliferation were observed only in gastric cancer cell lines expressing USP12^WT^, not in cells expressing USP12^C48S^ (Fig. 3C). Moreover, overexpressing USP12^WT^ in gastric cancer cell lines enhanced cell migration and invasion, while overexpression of USP12^C48S^ did not (Fig. 3D, E). Additionally, only the overexpression of USP12^WT^ promoted cell cycle progression (Fig. 3F, G) and inhibited apoptosis (Fig. 3H, I), whereas USP12^C48S^ did not. This suggests that the promotion of gastric cancer progression by USP12 is dependent on its deubiquitinating enzyme function.

### USP12 regulates the Hippo/YAP pathway in human gastric cancer

**Fig. 4:**
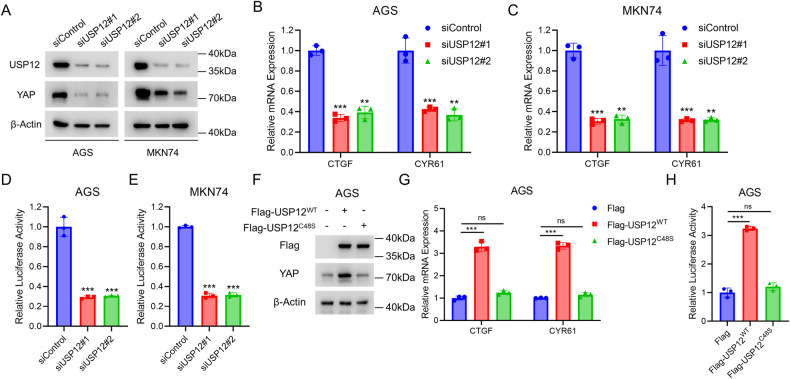
USP12 promotes Hippo/YAP signaling in gastric cancer. **A** Western blotting analysis showing USP12 depletion decreases YAP protein stability. AGS and MKN74 cells transfected with 50 nM siControl or two independent siUSP12. Cell lysates were immunoblotted with the indicated antibodies. β-Actin was used as internal control. **B**, **C** qRT-PCR analysis of YAP target genes (CTGF, CYR61) expression. AGS and MKN74 cells transfected with 50 nM siControl or two independent siUSP12 for 48 h. Total RNA was extracted for gene expression analysis. Each group was analyzed in triplicate. *P < 0.05; **P < 0.01; ***P < 0.001 for target gene expression comparison. Luciferase assays showing USP12 depletion affects TEAD-luciferase activity in AGS (**D**) and MKN74 (**E**) cells. Each group was analyzed in triplicate. *P < 0.05; **P < 0.01; ***P < 0.001 for luciferase activity comparison. **F** Western blotting analysis showing USP12^WT^ increases YAP protein stability but not USP12^C48S^. AGS cells were transfected with 1 μg Flag or Flag-USP12^WT^/Flag-USP12^C48S^. Cell lysates were immunoblotted with the indicated antibodies. β-Actin was used as internal control. **G** qRT-PCR analysis of YAP target genes (CTGF, CYR61) expression. AGS cells were transfected with 1 μg Flag or Flag-USP12^WT^/Flag-USP12^C48S^ for 48 h. Total RNA was extracted for gene expression analysis. Each group was analyzed in triplicate. *P < 0.05; **P < 0.01; ***P < 0.001 for target gene expression comparison. **H** Luciferase assays showing USP12^WT^ increases TEAD-luciferase activity but not USP12^C48S^ in AGS cells. Each group was analyzed in triplicate. *P < 0.05; **P < 0.01; ***P < 0.001 for luciferase activity comparison.

We further investigated the function of USP12 in Hippo/YAP signaling pathway in gastric cancer. Western blot analysis showed that the deletion of USP12 decreased levels of YAP protein in AGS and MKN74 cell lines (Fig. 4A). Considering that the Hippo signaling pathway functions as a kinase cascade signaling pathway, we examined the effects of knockdown USP12 on LATS1, phosphorylated LATS1, and phosphorylated YAP, components of the Hippo signaling pathway. Consequently, the depletion of USP12 had no impact on the levels of LATS1 protein, LATS1 phosphorylation, or YAP phosphorylation (Supplementary Fig. S1A). Furthermore, the YAP classical target genes CTGF and CYR61 were found to be downregulated following the deletion of USP12, consistent with the findings in Fig. 1 from our analysis of the TCGA database (Fig. 4B, C). According to the luciferase reporter assay in AGS and MKN74 cells, USP12 deletion inhibits TEAD response elements activity (Fig. 4D, E). Furthermore, we overexpressed USP12^WT^ and USP12^C48S^ in AGS cells to observe the effect of USP12 deubiquitinating enzyme activity on Hippo/YAP signaling. Western blotting results demonstrated that USP12^WT^ increased the protein level of YAP, whereas USP12^C48S^ did not have the same effect (Fig. 4F). Furthermore, only USP12^WT^ modulated the expression of YAP target genes (Fig. 4G). Moreover, USP12^WT^ was found to enhance the transcriptional activity of TEAD, whereas USP12^C48S^ did not demonstrate similar effects (Fig. 4H). Taken together, these findings indicate that USP12 can activate HIPPO/YAP signaling, with this effect being dependent on its deubiquitinating enzyme activity.

### USP12 promotes gastric cancer progression via the Hippo/YAP Axis

**Fig. 5:**
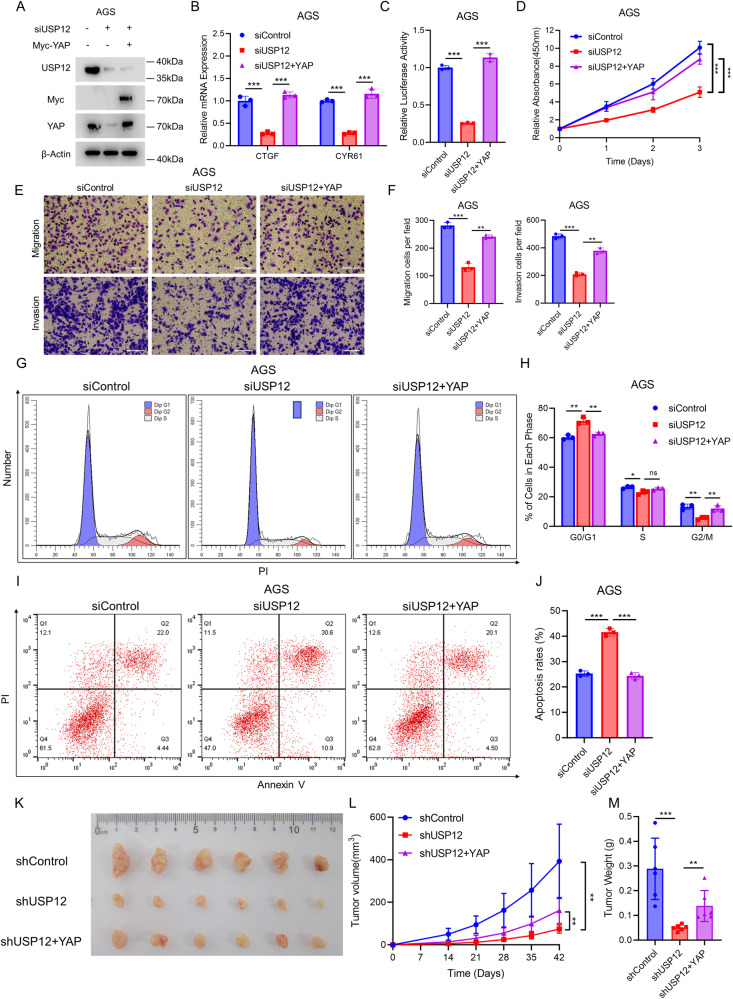
YAP rescues gastric cancer cell suppression induced by USP12 deletion. **A** Western blotting analysis showing USP12 depletion decreases YAP protein stability could be rescued by overexpression of YAP. AGS cells transfected with 50 nM siControl or siUSP12, and after 24 h, transfected with 0.5 μg Myc or Myc-YAP. Cell lysates were immunoblotted with the indicated antibodies. β-Actin was used as internal control. **B** qRT-PCR analysis of YAP target genes (CTGF, CYR61) expression. AGS cells transfected with 50 nM siControl or siUSP12 for 48 h, and after 24 h, transfected with 0.5 μg Myc or Myc-YAP. Total RNA was extracted for gene expression analysis. Each group was analyzed in triplicate. *P < 0.05; **P < 0.01; ***P < 0.001 for target gene expression comparison. **C** Luciferase assays showing USP12 depletion affects TEAD-luciferase activity and its could be rescued by YAP expression. Each group was analyzed in triplicate. *P < 0.05; **P < 0.01; ***P < 0.001 for luciferase activity comparison. **D** USP12 depletion inhibits the proliferation of gastric cancer cells could be rescued by overexpression of YAP. AGS cells transfected with 50 nM siControl or siUSP12 for 48 h, and after 24 h, transfected with 0.5 μg Myc or Myc-YAP. After 24 h, CCK-8 assay was used to determine cell absorbance at the indicated time points after transfection. Experiments were performed in triplicate. *P < 0.05; **P < 0.01; ***P < 0.001 for cell growth comparisons. **E**, **F** USP12 depletion inhibits the migration of gastric cancer cells could be rescued by overexpression of YAP. AGS cells transfected with 50 nM siControl or siUSP12 for 48 h, and after 24 h, transfected with 0.5 μg Myc or Myc-YAP. After 24 h, cells were separately plated into the Transwell chambers. For invasion experiments, it is necessary to spread the matrix gel before planting the cells. After 12 h, the cells were fixed and stained with crystal violet. The transwell cell number was determined to indicate cell migration (upper) and invasion (lower) activity (**E**). Quantification of transwell results using ImageJ software (**F**). Scale bar 100 μm. *N* = 3, *P < 0.05; **P < 0.01; ***P < 0.001 for cell migration comparisons. **G**, **H** USP12 depletion inhibits cell cycle progression, and it could be rescued by overexpression of YAP. AGS cells transfected with 50 nM siControl or siUSP12 for 48 h, and after 24 h, transfected with 0.5 μg Myc or Myc-YAP. After 24 h, the cells were harvested, fixed with 70% ethanol, and stained with propidium iodide. The cells were subjected to FACS analysis (**G**). Quantitative summary of cell-cycle analysis using graphpad software (**H**). Experiments were performed in triplicate. *P < 0.05; **P < 0.01; ***P < 0.001 for cell proportion comparisons. **I**, **J** USP12 depletion inhibits cell apoptosis, and it could be rescued by overexpression of YAP. AGS cells transfected with 50 nM siControl or siUSP12 for 48 h, and after 24 h, transfected with 0.5 μg Myc or Myc-YAP. After 24 h, the cells were harvested, and stained with Annexin V-PI (**I**). Quantitative summary of apoptosis analysis using graphpad software (**J**). Experiments were performed in triplicate. *P < 0.05; **P < 0.01; ***P < 0.001 for cell proportion comparisons. **K**–**M** USP12 depletion inhibits the tumor growth of AGS cells in the xenograft model, it could be rescued by overexpression of YAP. Harvested and photographed tumors in the shControl, shUSP12 and shUSP12+YAP (**K**), tumor volume (**L**) and weight (**M**) growth in each mouse. *P < 0.05; **P < 0.01; ***P < 0.001.

To investigate the possible role of Hippo/YAP axis in the progression of gastric cancer induced by USP12, rescue experiments were performed. Western blotting analysis revealed that YAP overexpression successfully rescued the reduced YAP protein levels, which had resulted from USP12 deletion (Fig. 5A). Furthermore, qRT-PCR and luciferase assays indicated that YAP overexpression was able to reverse the down-regulation of Hippo target genes (Fig. 5B) and the decrease in TEAD transcriptional activity caused by USP12 deletion (Fig. 5C). We conducted phenotypic rescue experiments to further investigate the impact of USP12 deletion on gastric cancer cells. By the CCK8 assay, it was observed that the proliferation of gastric cancer cells was suppressed by USP12 deletion, and this inhibition was partially mitigated by YAP overexpression (Fig. 5D). Moreover, the transwell assay showed that overexpression of YAP partially restored the cell migration and invasion suppressed by USP12 deletion (Fig. 5E, F). Additionally, flow cytometry analysis using PI staining revealed that YAP overexpression partially rescued the G0/G1 arrest induced by USP12 deletion (Fig. 5G, H). Besides, flow cytometry apoptosis Annexin V-PI staining assay indicated that USP12 deletion promoted apoptosis, and YAP overexpression was able to rescue this effect (Fig. 5I, J). Furthermore, depletion of USP12 effectively inhibited gastric cancer tumor growth in vivo in a xenograft model, and overexpression of YAP rescued this effect (Fig. 5K–M). These findings collectively highlight the intricate interplay between USP12 and YAP in the regulation of gastric cancer cell phenotypes.

### USP12 interacts with YAP and regulates YAP protein stability via the proteasome pathway

**Fig. 6:**
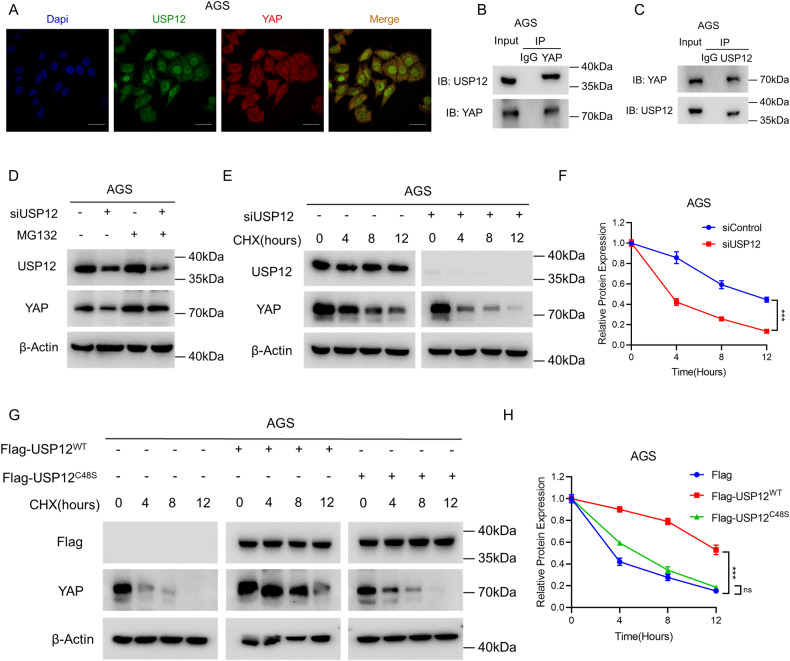
USP12 interacts with YAP proteins and regulates YAP protein stability via the proteasome pathway. **A** Immunofluorescence staining assay showing the localization patterns of USP12 and YAP in AGS cells. Intracellular localization of USP12 (green) and YAP (red) is shown. Nucleus (blue) were stained with DAPI. Scale bar, 20 µm. **B**, **C** Co-IP showing the endogenous interaction between USP12 and YAP. Llysates of AGS cells were precipitated with anti-YAP or anti-USP12 antibodies, and the precipitates were examined by immunoblotting. **D** Immunoblot analysis showing the expression level of YAP protein in siControl and siUSP12 expressing AGS cells by treat with 10 μM proteasome inhibitor MG132. **E**, **F** Immunoblot analysis showing USP12 deletion decreased YAP half-life. siControl and siUSP12 were transfected in AGS cells. Cells were treated with 100 μM cycloheximide (CHX) for the indicated times. Cell lysates were immunoblotted with the indicated antibodies. β-Actin was used as internal control (**E**). The expression of YAP protein was estimated by ImageJ software and is represented graphically (**F**). Experiments were performed in triplicate. *P < 0.05; **P < 0.01; ***P < 0.001. **G**, **H** USP12 deubiquitinating enzyme inactivity mutant cannot increase YAP half-life. AGS cells were transfected with 1 μg Flag or Flag-USP12^WT^/Flag-USP12^C48S^ for 24 h. Then cells were treated with 100 μM cycloheximide (CHX) for the indicated times. Cell lysates were immunoblotted with the indicated antibodies. β-Actin was used as internal control (**G**). The expression of YAP protein was estimated by ImageJ software and is represented graphically in the right panel (**H**). Experiments were performed in triplicate. *P < 0.05; **P < 0.01; ***P < 0.001.

We further investigated the potential mechanism of action of USP12 in regulating gastric cancer progression through the Hippo/YAP pathway. Endogenous immunofluorescence analysis revealed that USP12 and YAP co-localized in both the cytoplasm and nucleus (Fig. 6A). Further, we found that knockdown USP12 did not alter YAP cellular localization by immunofluorescence experiments (Supplementary Fig. S2A). Additionally, the Co-IP assay confirmed the interaction between USP12 and YAP (Fig. 6B, C). As YAP degradation is known to depend on the action of the proteasome, we subsequently examined whether USP12, as a deubiquitinating enzyme, regulates YAP protein via the proteasomal pathway. Treatment with the proteasome inhibitor MG132 demonstrated that YAP protein levels reduced by USP12 deletion, and MG132 treatment mitigates this reduction in YAP protein (Fig. 6D). Furthermore, CHX analysis of the half-life of YAP protein revealed a significant reduction in YAP protein half-life following USP12 deletion (Fig. 6E, F). We then investigated the effect of USP12 overexpression on YAP protein half-life and found that USP12 overexpression increased YAP protein stability, whereas USP12 enzyme inactivation mutants USP12^C48S^ did not (Fig. 6G, H). As a result of these findings, we have concluded that USP12 is interacting with YAP protein and regulating the stability of YAP protein through the proteasome pathway.

### USP12 stabilizes YAP protein by inhibiting K48-linked polyubiquitination at the YAP K315 site

**Fig. 7:**
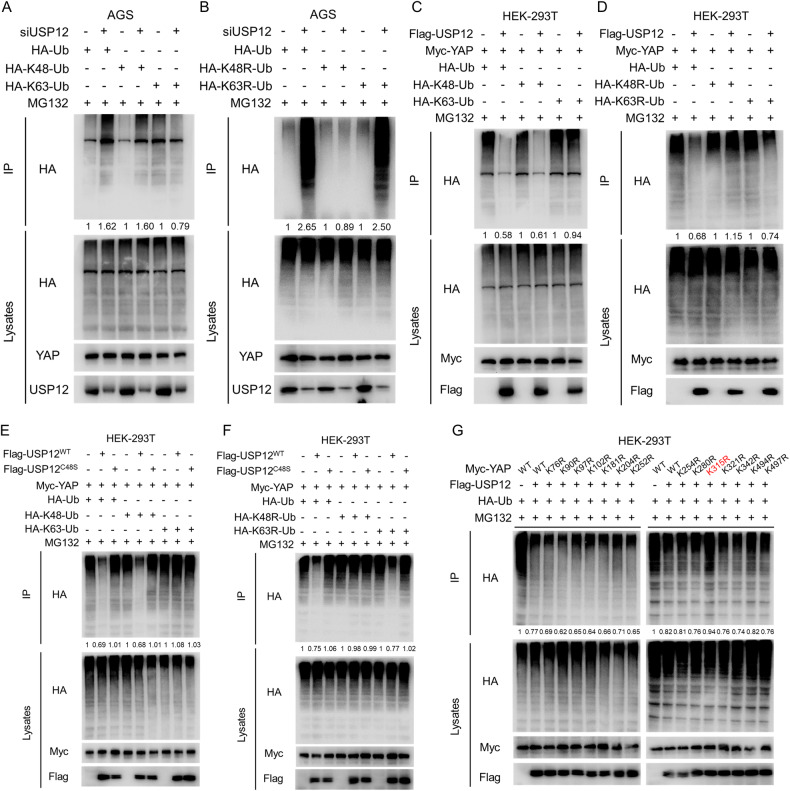
USP12 stabilizes YAP via inhibiting YAP K48-linked poly-ubiquitination of the YAP K315 site. **A**, **B** Depletion of USP12 increased total ubiquitination and K48-linked poly-ubiquitination of the YAP. AGS cells were transfected with 0.5 µg HA-Ub/HA-K48/HA-K63 Ub plasmid or 0.5 µg HA-Ub/HA-K48R/HA-K63R Ub plasmid and 50 nM USP12 siRNA upon 10 µM MG132 treatment for 6 h. Cells were lysed and co-immunoprecipitated with anti-YAP antibody, and immunoblotted with the indicated antibodies. **C**, **D** USP12 Deubiquitinates YAP via K48-linked polyubiquitination. HEK-293T cells were transfected with 0.5 µg HA-Ub/HA-K48/HA-K63 Ub plasmid or 0.5 µg HA-Ub/HA-K48R/HA-K63R Ub plasmid, 1 µg Flag-USP12 and 1 µg Myc-YAP upon 10 µM MG132 treatment for 6 h. Cells were lysed and co-immunoprecipitated with anti-Myc antibody, and immunoblotted with the indicated antibodies. **E**, **F** USP12^WT^ can be deubiquitinated YAP by K48-linked polyubiquitination, whereas USP12^C48S^ cannot. HEK-293T cells were transfected with 0.5 µg HA-Ub/HA-K48/HA-K63 Ub plasmid or 0.5 µg HA-Ub/HA-K48R/HA-K63R Ub plasmid, 1 µg Flag-USP12^WT^/Flag-UAP12^C48S^ and 1 µg Myc-YAP upon 10 µM MG132 treatment for 6 h. Cells were lysed and co-immunoprecipitated with anti-Myc antibody, and immunoblotted with the indicated antibodies. **G** USP12 ubiquitinates YAP through the K315 site of YAP. HEK-293T cells were transfected with 0.5 µg HA-Ub plasmid, 1 µg Flag-USP12 and 1 µg Myc-YAP WT/K76R/K90R/K97R/K102R/K181R/K204R/K252R/K254R/K280R/K315R/K321R/K342R/K494R/K497R upon 10 µM MG132 treatment for 6 h. Cells were lysed and co-immunoprecipitated with anti-Myc antibody, and immunoblotted with the indicated antibodies.

As USP12 is a deubiquitinating enzyme, we further investigated its impact on YAP ubiquitination. Endogenous ubiquitination experiments showed that USP12 deletion resulted in increased total ubiquitination and K48-linked polyubiquitination. However, polyubiquitination of the K63-inked could not increase as a result of USP12 deletion (Fig. 7A). Moreover, the dominant negative mutant K63 ubiquitin (K63R) was observed to increase the impact of USP12 on YAP polyubiquitination, while K48R is unable to deubiquitinate YAP (Fig. 7B). Similarly, overexpression of USP12 resulted in the inhibition of total ubiquitination and K48-linked polyubiquitination of YAP proteins, while polyubiquitination of the K63-inked was unaffected (Fig. 7C). Moreover, the overexpression of USP12 was observed to inhibit K63R-linked polyubiquitination, while it did not affect K48R (Fig. 7D). Besides, we performed cell nuclear-cytoplasmic separation after overexpression of USP12, and the lysates obtained from the isolation were subjected to ubiquitination experiments. The results showed that the deubiquitination of YAP by USP12 was observed in both nucleus and cytoplasm (Supplementary Fig. S3A). These results suggest that USP12 may stabilize the YAP protein through K48-linked polyubiquitination. Further ubiquitination experiments demonstrated that only the USP12^WT^ could deubiquitinate YAP proteins, while the mutant USP12^C48S^, lacking deubiquitinating enzyme activity, could not (Fig. 7E, F). To further elucidate the mechanism by which USP12 deubiquitinates YAP, we investigated the specific site on the YAP protein targeted by USP12. Given that the YAP protein has 14 lysine sites for ubiquitin attachment, we created lysine mutant forms. Ubiquitination assays demonstrated that USP12 inhibits polyubiquitination of the K315 site of YAP (Fig. 7G). Taken together, ubiquitination assays demonstrated that USP12 inhibits polyubiquitination of the K315 site of YAP.

## Discussion

**Fig. 8:**
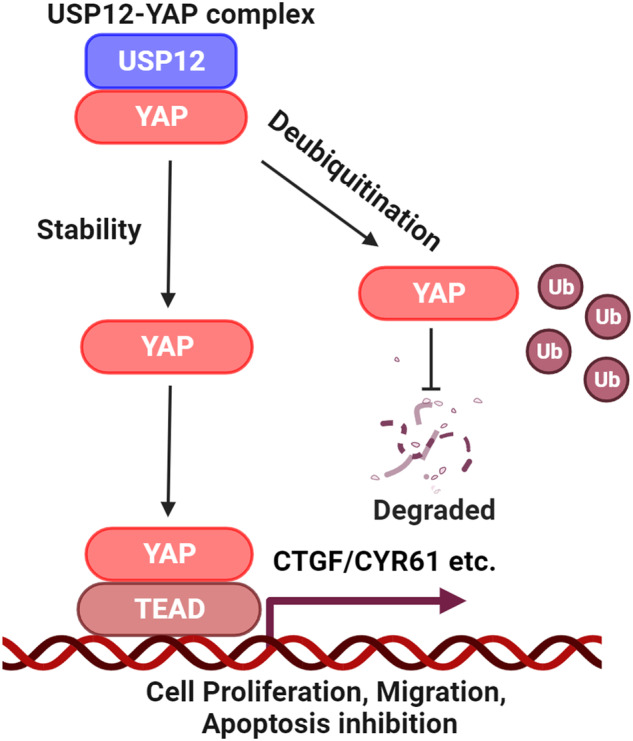
A hypothetical model of the mechanism of USP12 regulation of Hippo/YAP signaling in gastric cancer. USP12 interacts with YAP to inhibit K48-linked ubiquitination at the K315 site, stabilizing the YAP protein and promoting transcription of downstream target genes, which contributes to gastric cancer progression.

In the currently study, we performed siRNA screening and identified that USP12 is essential for controlling the stability of the YAP protein in gastric cancer. Clinical data indicated a correlation between USP12 expression and the YAP gene signature. Furthermore, gastric cancer patients with high expression of USP12 exhibit a poor survival. USP12 activation facilitates gastric cancer progression by modulating YAP stability. Mechanistically, USP12 could interact with YAP to suppress K48-linked poly-ubiquitination, predominantly at K315 site (Fig. 8). Our research uncovers a novel mechanism of post-translational regulation for Hippo signaling, identifying USP12 as a promising target for therapeutic intervention in gastric cancer.

The clinical value of therapeutic targeting Hippo/YAP axis is widely acknowledged in cancer field, while quite a lot of researches started to discovery novel inhibitors, which aimed to inhibit Hippo-driven cancers, such as hepatocarcinoma and gastric cancer. In previous studies, verteporfin could inhibit the binding of YAP to TEAD, which subsequently inhibited its target gene expression and cancer progression [23]. However, further clinical trials showed several shortcomings of verteporfin, such as instable to light exposure and cellular toxicity by ROS (reactive oxygen species) [24]. Further researches identified VGLL4 could compete with YAP for TEAD binding, which blocked Hippo target gene expression and tumor growth [25]. The VGLL4-like peptide Super-TDU was applied in vivo, which implicated the inhibition effect in gastric tumor growth [14]. However, all these potential inhibitors are still in pre-clinical trials. Besides, we have to notice that the penetration capability to cross cell membrane and the stability of the peptide or inhibitors might be outstanding issues, which needed to be overcome before clinical use. Thus, we concluded that there is no mature Hippo targeting drugs in clinical cancer treatment.

The USP (Ubiquitin-proteasome system) plays a vital role in regulating protein stability, degradation, and function. There are several modes of poly-ubiquitination, including ubiquitination of lysine sites 9, 11, 27, 29, 33, 48, 63 and linear ubiquitination [26]. Among them, the K48-linked ubiquitination was the most thoroughly investigated, which always linked to protein degradation, while the other forms of ubiquitination could link to protein trafficking, protein function change or increased protein stability [27]. Ubiquitination is a cascade process in which ubiquitin-activating enzyme E1, ubiquitin-conjugating enzyme E2 and ubiquitin ligases E3 play important roles. Deubiquitinating enzymes counteract poly-ubiquitination by catalyzing a deubiquitination process. It has been demonstrated that some deubiquitinases can modulate the stability of YAP and promote the progression of gastric cancer in several recent studies. Nevertheless, these studies are based on the siRNA screening in HEK293 cells [18, 21]. In our study, we did the siRNA screening from one typical gastric cancer cell line AGS cells, and identify crucial deubiquitinases that affect the progression of gastric cancer and are potentially significant for treatment.

USP12 (Ubiquitin Specific Peptidase 12) was firstly discovered as a novel UBH (Ubiquitin homology) domain protein in 1998, which was expressed in retina, neuronal ganglia and gut [28]. Further studies demonstrated that USP12 could be a coactivator for androgen receptor and its deubiquitinase activity depended on the existence of WDR20 and WDR48 [29]. USP12 is involved in multiple biological processes, such as immune responses against viruses, autophagy, and cell death. Studies have demonstrated that USP12 facilitates in the advancement of cancer by deubiquinating substrates. For example, USP12 could deubiquitinate RRM2 and promote non-small cell lung cancer progression [30]. In prostate cancer, USP12 could also modulate P53-MDM2-AR-AKT signaling [31]. It remains unclear how USP12 contributes to gastric cancer. Our research uncovered a new functional connection between USP12 and YAP, suggesting that USP12 could potentially be a valuable target for drug development in the management of gastric cancer.

In conclusion, we confirmed USP12 as an oncogene in both in vitro and in vivo models of gastric cancer. Our study showed that the expression of USP12 was increased in gastric cancer. Furthermore, it was discovered that the elevated expression of USP12 was associated with poor survival specifically in gastric cancer patients. Besides, USP12 interacted with YAP protein, suppressing YAP poly-ubiquitination by K48-linked and preventing proteasome-mediated degradation in gastric cancer cells. Therefore, targeting USP12 activity or gene expression could provide an attractive strategy for treating gastric cancer.

## Materials and methods

### Cell culture

The HEK-293T cells and AGS cells were acquired from American Type Culture Collection (ATCC). The human gastric cancer cell MKN74 cells were purchased from Keycell Biotechnology (China). Authentication of cell lines was performed by analyzing STR profiles with the Promega Power Plex 21 system. AGS and MKN74 cells were cultured with RPMI-1640 (R8758, Sigma-Aldrich) and HEK-293T cells were cultured with DMEM (D6429, Sigma-Aldrich). Cells were supplemented with 10% fetal bovine serum (10270-106, Gibco) and 1% Penicillin-Streptomycin-Gentamicin Solution (C0223, Beyotime) at 37 °C in an environment with 5% CO_2_. For Cell cryopreservation, cells were suspended in serum-free cell cryopreservation solution (C40100, New Cell Molecular Biotech).

HEK-293T cells were transduced with shRNA lentiviral particles to produce lentiviruses that target USP12 for depletion. The pMD2G and psPAX2 envelope plasmids were co-transfected into the cells, resulting in the acquisition of the lentivirus after 2 days. Subsequently, AGS cells were treated with 2 mL of medium without antibiotics, which included 200 μL of the lentivirus. Infected cells were then screened with 2 μg/mL puromycin (Merck Millipore) to generate stable cell lines.

### DUB siRNA library screening

AGS cells were transfected with various siDUBs obtained from a siRNA collection containing 98 human DUBs acquired from Human Deubiquitinating Enzyme in the Dharmacon siRNA Library (GU-104705). Following a 48-hour period, RNA was isolated and converted into cDNA through reverse transcription. The expression levels of the CTGF gene, which is a downstream target of YAP in the Hippo signaling pathway, were assessed to identify potential regulation. The research concentrated on the deubiquitinating enzyme USP12 based on the preliminary results obtained.

### Mouse xenograft tumor model

For the in vivo xenograft tumor model, we used the 5-week-old female BALB/c nude mice obtained from SPF Biotechnology Co.,Ltd. The BALB/c nude mice were randomly divided into groups of 6 mice each for the experiment. Then each mouse was injected subcutaneously 4 × 10^6^ cancer cells suspended in 150 μL of PBS. For the following six weeks, mice were observed for tumor development. The volume of the tumor was subsequently determined using the prescribed formula: tumor volume = length × width^2^/2. All experimental procedures involving mice followed the ethical guidelines approved by the Ethics Committee of the Second Hospital of Shandong University.

### Plasmids and siRNA

The Flag-USP12, and Flag-USP12^C48S^ constructs were generated by PCR and then inserted into the pcDNA 3.1 vector. The Myc-YAP, Myc-YAP mutant contains mutations at residues (K76, K90, K97, K102, K181, K204, K252, K254, K280, K315, K321R, K342, K494, K497). Previous research utilized the HA-Ub, HA-K48, HA-K48R, HA-K63 and HA-K63R plasmids. Plasmids were transfected using Lipofectamine 2000 (1662298, Invitrogen). The RNA iMAX reagent (13778150, invitrogen) was used for the transfection of siRNA. We employed siRNAs (small interfering RNAs) to knock down USP12. The USP12 siRNA sequences were siRNA#1: GAG CCU UCU UAC AUG CUU A; siRNA#2: GCA GUG AAU ACA CGU AUU A; Nagative control: UUC UCC GAA CGU GUC ACG U. The USP12 shRNA sequences were: CAT CAA TTA CTC ACT GCT TAA; shControl sequences were: TTC TCC GAA CGT GTC ACG T.

### Luciferase reporter assay

The cells were transfected with 50 μM siUSP12 or siControl, TEAD luciferase reporter plasmid, and Renila. The cells were harvested after 24 h, and used for luciferase assay (Dual-Luciferase^®^ Reporter Assay System, E1090, Promega).

### RNA extraction and real-time PCR

RNA isolater Total RNA Extraction Reagent (R401, Vazyme Biotech Co.,Ltd) was used to extract total RNA. Then the total RNA reverse transcribed to cDNA, and amplified using the gene specific primers and SYBR GREEN (AceQ Universal SYBR qPCR Master Mix, Q511, Vazyme Biotech Co.,Ltd) on the 7500 real-time quantitative PCR system. Internal control was used by 36B4. Primers used were as follows: USP12: 5′-AGT TTC CGG TCA ATG AGC ACT-3′ (Forward); 5′-TCC CGA AAT GGA CGA CAA AAA T-3′ (Reverse). CTGF: 5′-CAG CAT GGA CGT TCG TCT G-3′ (forward); 5′- AAC CAC GGT TTG GTC CTT GG-3′ (reverse). CYR61: 5′-GGT CAA AGT TAC CGG GCA GT-3′ (forward); 5′- GGA GGC ATC GAA TCC CAG C-3′ (reverse). 36B4: 5’-CGA CCT GGA AGT CCA ACT AC-3’ (forward); 5′-ATC TGC TGCA TCT GCT TG-3′ (reverse).

### Western blotting

The cells were collected and then lysed cells using RIPA buffer (P0013B, Beyotime) with phosphatase inhibitor (Biomake, B15001) and protease inhibitor cocktail, (Biomake, B14001). Protein samples were loaded on SDS/PAGE gels, separated, and then transferred protein to PVDF membranes (IPVH00010, Merk millipore). Following the blocking of the membrane using a solution of 5% skim milk powder in 5% Tween-20 PBST for 1 h at room temperature, the membrane was incubated with the appropriate antibody overnight at 4 °C. After being washed three times with PBST and the membrane was then probed with the secondary antibody. After washing the membrane three more times with PBST, the membrane was developed using the ECL system (Bio-rad ChemiDoc). The antibodies were as follows: anti-β-Actin antibody (Cell signaling technology, 4967S), anti-Flag antibody (Sigma, F1804), anti-USP12 antibody (Proteintech, 12608-1-AP), anti-Myc antibody (Proteintech, 16286-1-AP), anti-YAP antibody (Cell signaling technology, 14074S), anti-Phospho-YAP antibody (Cell signaling technology, 13008S), anti-Phospho-LATS1 (Thr1079) antibody (Cell signaling technology, 8654S), anti-LATS1 antibody (Cell signaling technology, 3477S), and anti-HA antibody (Biolegend, 90513). Goat Anti-Rabbit IgG (Beyotime, A0208) or Goat Anti-Mouse IgG (Beyotime, #A0216) were used for detection, and ECL Kit was used to visualize the signals (Meilunbio, MA0186).

### CCK8 assay

After silencing USP12 for 24 h, both siUSP12 and siControl cells were plated at 5000 cells per well in 96-well plates. CCK8 reagent was added at 0, 1, 2, and 3 days, followed by 1 h incubation at 37 °C with 5% CO_2_. Cell absorbance at 450 nm was then determined.

### Transwell assay

After USP12 knockdown for 24 h, siControl and siUSP12 cells were separately plated into the Transwell chambers (Corning, 3422). Next, 50,000 cells were suspended in 200 μL of medium without serum, and the chambers were placed in 500 μL medium containing 20% serum. For invasion experiments, it is necessary to spread the matrix gel before planting the cells. The cells were fixed and stained with crystal violet after a 12-h period (Beyotime, C0121).

### PI staining

For siUSP12 knockdown experiments, AGS and MKN74 cells were transfected with siControl or siUSP12. After 24 h, cells were collected and resuspended into a single-cell suspension. The cells were washed three times with PBS, fixed in 70% anhydrous ethanol for 1 h, and stained with propidium iodide (Invitrogen, FxCycle PI/RNase Staining Solution, F10797). Fluorescence intensity was quantified by employing BD Flow cytometry.

### Annexin V-PI staining

For siUSP12 knockdown experiments, AGS and MKN74 cells were transfected with siUSP12 or siControl. Cells were collected after 24 h and resuspended into a single-cell suspension. After being washed three times with PBS, the cells stained with Annexin V-PI apoptosis staining kit (BD,556547). Fluorescence intensity was quantified by employing BD LSR flow cytometry.

### Immunofluorescence assay

The AGS cells were fixed with 4% paraformaldehyde for 10 min and permeabilized 0.2% Triton X-100 (T8200, Solarbio) for 10 min at room temperature. Subsequently, the cells were blocked with PBS supplemented with 5% BSA (ST025, Beyotime) for 1 h. AGS cells were incubated overnight at 4 °C with rabbit anti-USP12 polyclonal antibody (PA5-139844, Invitrogen) and mouse anti-YAP antibody (SC-110199, Santa Cruz). Then the following day, cells were incubated at room temperature for 1 h using Goat anti-Mouse IgG (H + L) Alexa Fluor™ Plus 555 (Invitrogen, 32727) for mouse antibody and Goat anti-Rabbit IgG (H + L) Alexa Fluor™ Plus 488 (Invitrogen, 32731) for rabbit antibody. Three washes later, the cells were stained with DAPI (Beyotime, C1005) to visualize the cell nucleus. The images were captured with a confocal laser scanner and further processed and analyzed using ImageJ.

### Co-Immunoprecipitation assay

Cells were collected using Co-IP cell lysis buffer (P0013, Beyotime) with added protease inhibitors. The cell lysate was then centrifuged at 12,000 × *g* for 30 min at 4 °C to collect the supernatant. The supernatant was incubated overnight at 4 °C with the desired antibody or IgG, along with protein A/G agarose (P2051/P2053, Beyotime). The next day, the combination was centrifuged at 3000 × *g* for 10 min at 4 °C, and then washed three times with lysis buffer, and the supernatant was discarded. Then add 2x SDS-PAGE loading buffer, heat at 99 °C for 10 min, and proceed on immunoblotting.

### Poly-ubiquitination detection assay

As an example of total poly-ubiquitination. Co-transfected of cells was performed with 2 µg of Myc-USP12 or Myc-tag and 0.5 µg of HA-Ubi plasmids, along with 0.5 µg of Flag-YAP, for a duration of 24 h to observe poly-ubiquitination of YAP. Subsequently, protein extraction was conducted 6 h after treatment with 10 μM MG132. Pre-clearance was initially conducted with 30 µL of protein A agarose (P2051, Beyotime) for a duration of 2 h. Following this step, the extract was incubated overnight with an anti-Flag antibody. Then the total polyubiquitinated YAP was detected using an anti-HA antibody through western blotting.

### TCGA data analysis and KMplot survival analysis

Information regarding the presence of USP12 in gastric cancer specimens is available through the TCGA database on the GDC data portal (https://portal.gdc.cancer.gov/). The analysis and counting of the obtained data were conducted using GraphPad Prism 8.0. Moreover, an analysis was conducted on 374 gastric cancer samples from the TCGA database to explore the relationship between USP12 and YAP target genes.

The study analyzed the relationship between USP12 expression and clinical prognosis through the KMPLOT database (https://kmplot.com).

### Statistics

All experiments, including CCK8, transwell, flow cytometry analysis, western blotting, qRT-PCR, and luciferase reporter assays, were independently conducted at least three times. Statistical analysis was carried out using GraphPad Prism. Data were presented as mean ± SD, and two-tailed Student’s *t* test was utilized for statistical analysis. Survival analysis was conducted with the Kaplan-Meier method and log-rank test. Differences were considered to be statistically significant when *P* < 0.05. *P < 0.05; **P < 0.01; ***P < 0.001

### Supplementary information

Supplementary Figure 1

Supplementary Figure 2

Supplementary Figure 3

Supplementary Figure Legends

Original Data File

## Data Availability

The original data for WB and qRT-PCR, as well as the siRNA screening data, are available in the supplementary materials, which also contain the authentication information for the cell lines.
