# Supplementary figures and images for: USP12 facilitates gastric cancer progression via stabilizing YAP
Source: Cell Death Discov. 2024 Apr 11;10:174. doi: 10.1038/s41420-024-01943-2 (PMC11009230; doi:10.1038/s41420-024-01943-2)

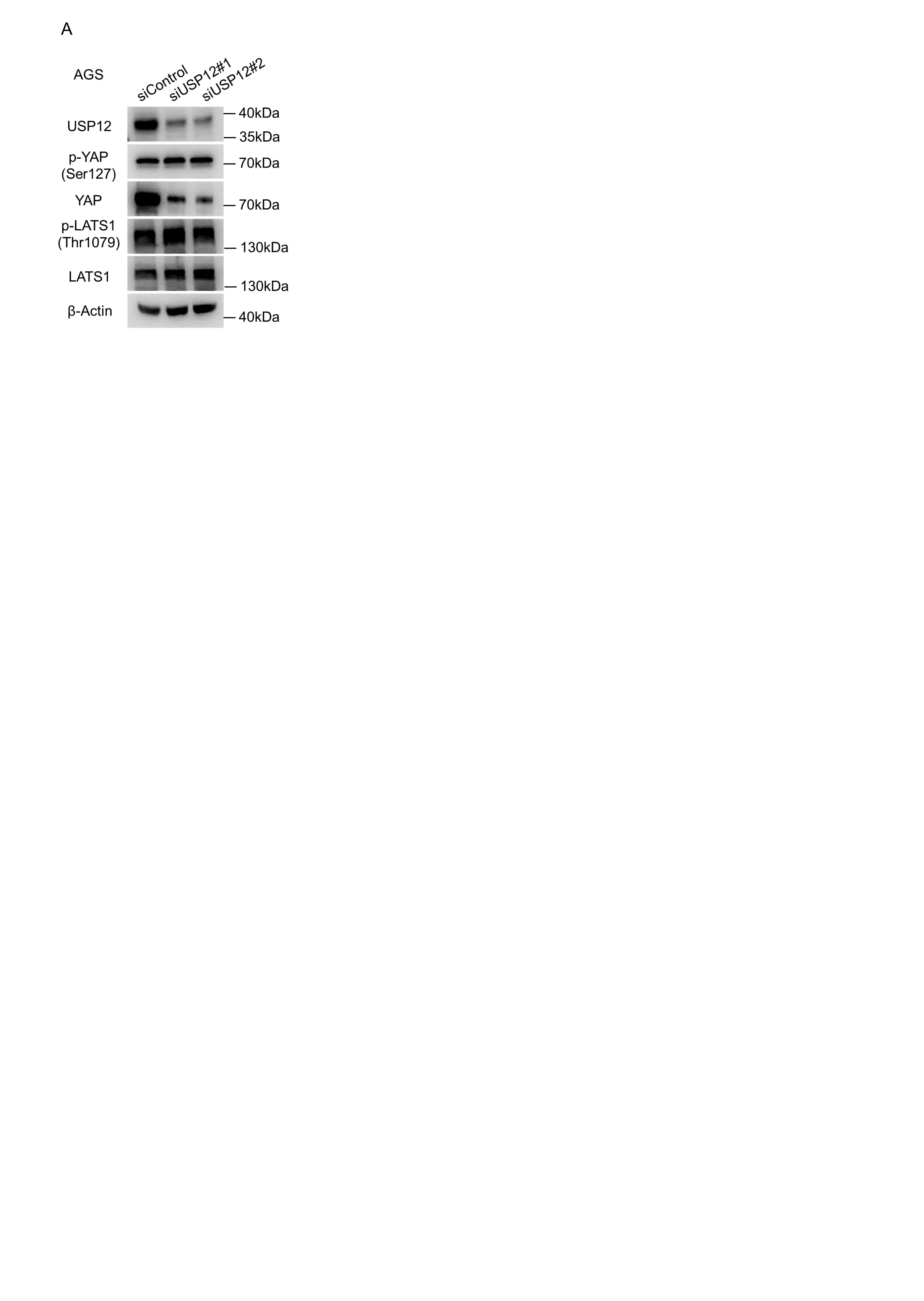

Supplement: Supplementary file 1 — Supplementary Figure 1 [file 41420_2024_1943_MOESM1_ESM.tif]

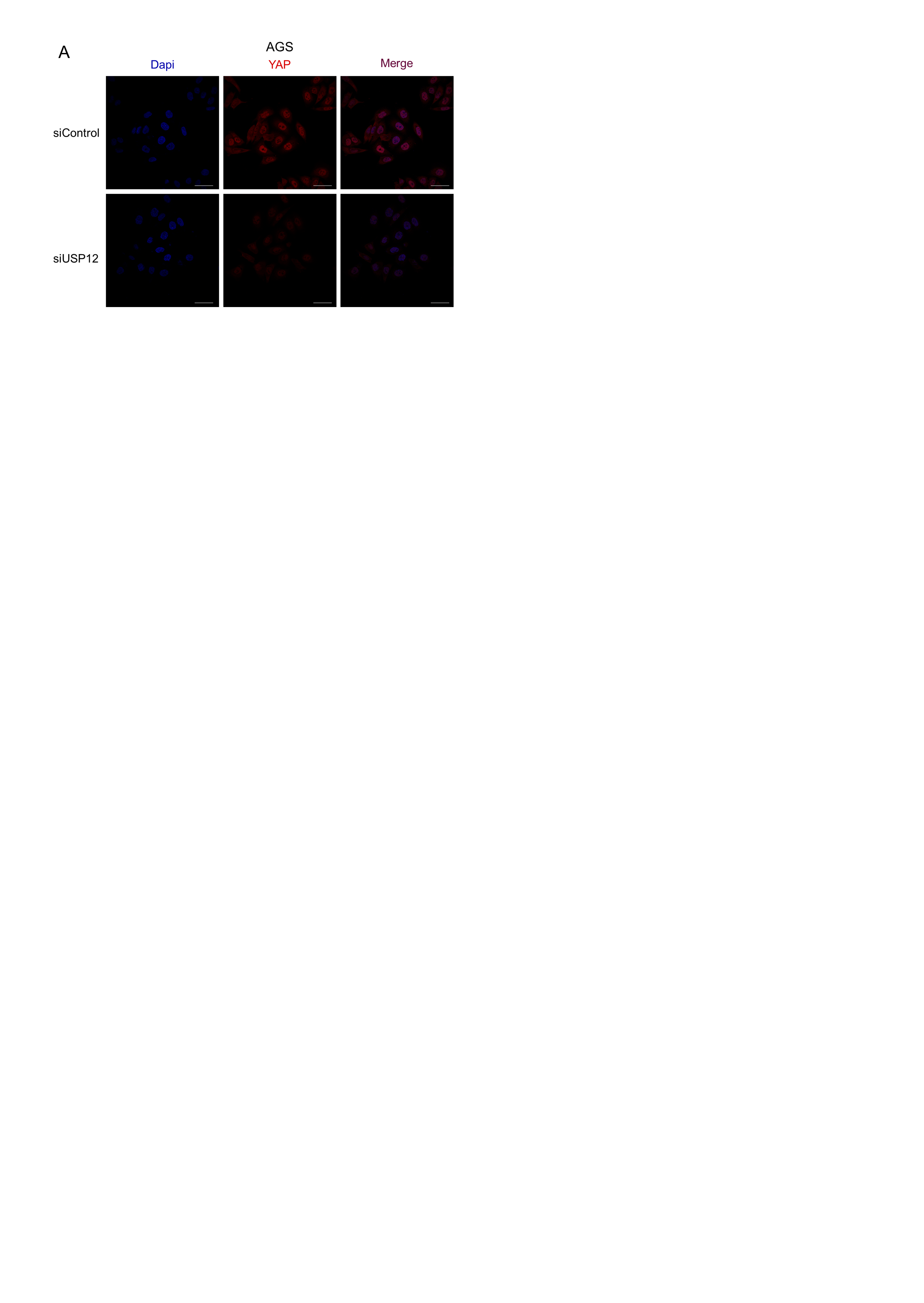

Supplement: Supplementary file 2 — Supplementary Figure 2 [file 41420_2024_1943_MOESM2_ESM.tif]

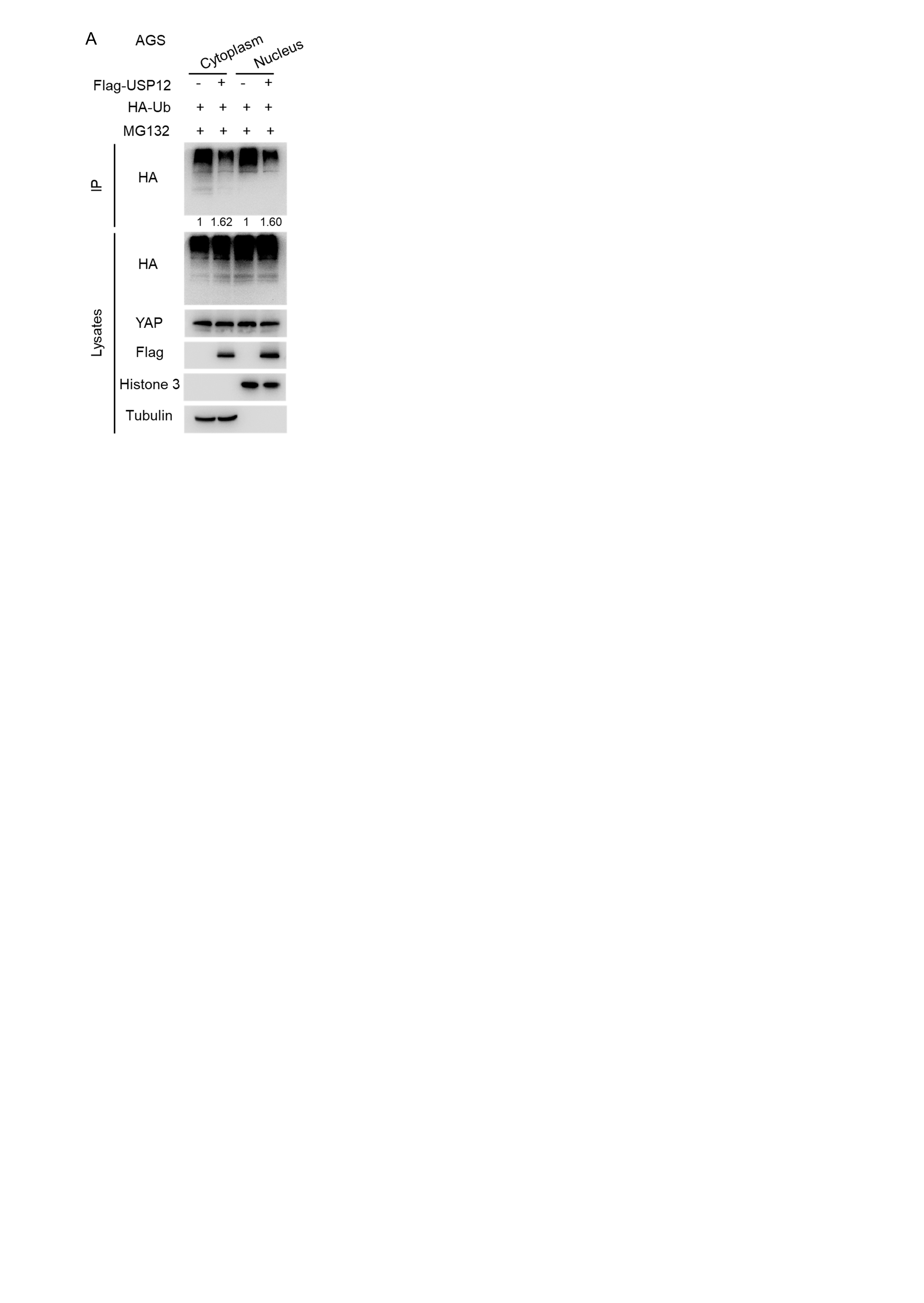

Supplement: Supplementary file 3 — Supplementary Figure 3 [file 41420_2024_1943_MOESM3_ESM.tif]
