# Supplementary material for: USP12 facilitates gastric cancer progression via stabilizing YAP
Source: Cell Death Discov. 2024 Apr 11;10:174. doi: 10.1038/s41420-024-01943-2 (PMC11009230; doi:10.1038/s41420-024-01943-2)
Supplement: Supplementary file 4 — Supplementary Figure Legends [file 41420_2024_1943_MOESM4_ESM.docx]

**Supplementary Figure Legends**

**Supplementary Figure 1. Depletion of USP12 did not affect LATS1, phosphorylation of LATS1, phosphorylation of YAP**

**A:** Western blotting analysis showing USP12 depletion decreases YAP protein stability but not LATS1, phosphorylation of LATS1, phosphorylation of YAP. AGS transfected with 50 nM siControl or two independent siUSP12. Cell lysates were immunoblotted with the indicated antibodies. β-Actin was used as internal control.

**Supplementary Figure 2. USP12 does not affect intracellular shuttle of YAP**

**A:** AGS transfected with 50 nM siControl or siUSP12. After 24 hours, an immunofluorescence assay was performed. Immunofluorescence staining assay showing the localization patterns of YAP in AGS cells. Intracellular localization of YAP (red) is shown. Nucleus (blue) were stained with DAPI. Scale bar, 20µm.

**Supplementary Figure 3. Deubiquitination of YAP by USP12 occurs in the nucleus and cytoplasm of cells**

**A:** AGS transfected with 2 μg Flag or 2 μg Flag-USP12, and then treatment with MG132 for 6 hours. After 24 hours, cell nuclear-cytoplasmic separation were performed. Cells were co-immunoprecipitated with anti-YAP antibody, and immunoblotted with the indicated antibodies.
