# Supplementary material for: USP12 facilitates gastric cancer progression via stabilizing YAP
Source: Cell Death Discov. 2024 Apr 11;10:174. doi: 10.1038/s41420-024-01943-2 (PMC11009230; doi:10.1038/s41420-024-01943-2)

Fig. 2A

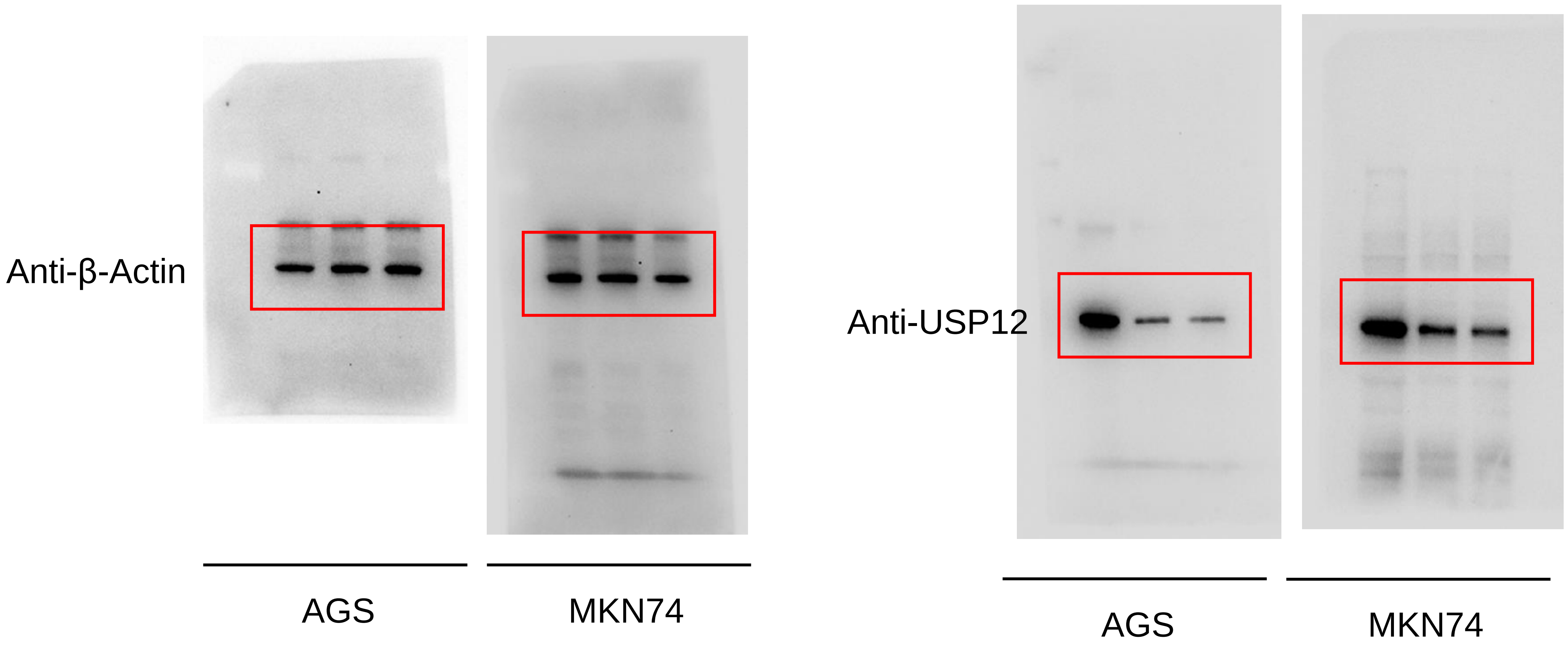

Fig. 2B

| Time (Days) | siControl | | | siUSP12#1 | | | siUSP12#2 | | |
|---|---|---|---|---|---|---|---|---|---|
| 0 | 1.346154 | 0.826923 | 0.826923 | 0.966443 | 1.006711 | 1.026846 | 0.870968 | 1.316129 | 0.812903 |
| 1 | 3.532692 | 3.225 | 3.936538 | 3.638255 | 2.973826 | 2.792617 | 2.607097 | 3.052258 | 2.839355 |
| 2 | 6.019231 | 6.096154 | 6.307692 | 4.006711 | 4.369128 | 4.651007 | 4.219355 | 3.425806 | 3.870968 |
| 3 | 12.59615 | 13.42308 | 13.59615 | 7.208054 | 7.348993 | 7.932886 | 6.445161 | 5.922581 | 5.825806 |

Fig. 2C

| Time (Days) | siControl | | | siUSP12#1 | | | siUSP12#2 | | |
|---|---|---|---|---|---|---|---|---|---|
| 0 | 0.9407 | 0.7881 | 1.2712 | 1.0392 | 1.1373 | 0.8235 | 0.9868 | 0.8882 | 1.125 |
| 1 | 4.1186 | 4.4492 | 4.8559 | 2.6275 | 2.3725 | 2.3137 | 2.0724 | 1.8947 | 2.7039 |
| 2 | 5.6695 | 5.3136 | 6.6356 | 3.2353 | 2.8824 | 3.8431 | 2.3487 | 3.4145 | 2.3882 |
| 3 | 9.9407 | 8.4661 | 9.7119 | 4.7255 | 5.5098 | 5.2157 | 4.4605 | 4.7763 | 5.0724 |

## Fig. 2E

| siControl | siUSP12#1 | siUSP12#2 |
|---|---|---|
| 408 | 123 | 118 |
| 411 | 144 | 102 |
| 396 | 119 | 105 |

| siControl | siUSP12#1 | siUSP12#2 |
|---|---|---|
| 360 | 134 | 88 |
| 341 | 117 | 92 |
| 332 | 124 | 101 |

## Fig. 2G

| siControl | siUSP12#1 | siUSP12#2 |
|---|---|---|
| 456 | 143 | 163 |
| 432 | 147 | 150 |
| 440 | 135 | 148 |

| siControl | siUSP12#1 | siUSP12#2 |
|---|---|---|
| 240 | 77 | 82 |
| 221 | 71 | 74 |
| 203 | 83 | 85 |

## Fig. 2J

| | siControl | | | siUSP12#1 | | | siUSP12#2 | | |
|---|---|---|---|---|---|---|---|---|---|
| G0/G1 | 55.62 | 54.35 | 53.17 | 65.34 | 64.12 | 67.03 | 63.16 | 66.11 | 62.64 |
| S | 29.19 | 30.11 | 31.09 | 28.08 | 28.41 | 26.54 | 26.77 | 24.76 | 27.22 |
| G2/M | 15.19 | 15.54 | 15.74 | 6.58 | 7.47 | 6.43 | 10.07 | 9.13 | 10.14 |

## Fig. 2K

| | siControl | | | siUSP12#1 | | | siUSP12#2 | | |
|---|---|---|---|---|---|---|---|---|---|
| G0/G1 | 59.73 | 58.14 | 57.35 | 66.2 | 67.15 | 68.74 | 64 | 67.48 | 68.07 |
| S | 31.57 | 32.33 | 31.86 | 27.73 | 26.77 | 27.05 | 29.55 | 25.87 | 24.83 |
| G2/M | 9 | 9.53 | 10.79 | 6.07 | 6.08 | 4.21 | 6.45 | 6.65 | 7.1 |

Fig. 2N

| siControl | siUSP12#1 | siUSP12#2 |
|---|---|---|
| 19.6 | 36.85 | 29.93 |
| 18.5 | 34.22 | 32.08 |
| 16.2 | 33.49 | 33.15 |

Fig. 2O

| siControl | siUSP12#1 | siUSP12#2 |
|---|---|---|
| 22.36 | 36.62 | 38.4 |
| 23.55 | 34.16 | 36.74 |
| 20.74 | 37.02 | 35.86 |

# Fig. 2Q

| Time (Days) | shControl | | | | | | shUSP12 | | | | | |
|---|---|---|---|---|---|---|---|---|---|---|---|---|
| 0 | 0 | 0 | 0 | 0 | 0 | 0 | 0 | 0 | 0 | 0 | 0 | 0 |
| 14 | 144.4247 | 77.24322 | 71.89301 | 82.97138 | 65.10514 | 74.60841 | 43.49667 | 44.51371 | 27.72047 | 38.17615 | 7.52895 | 4.38 |
| 21 | 251.0845 | 134.6481 | 131.4447 | 150.177 | 123.3557 | 128.4825 | 85.25601 | 84.62418 | 58.6558 | 65.93535 | 16.42995 | 9.32908 |
| 28 | 443.7679 | 211.5753 | 210.036 | 237.2949 | 197.7116 | 199.5851 | 140.3791 | 137.4497 | 98.29016 | 119.0894 | 29.7685 | 15.61581 |
| 35 | 842.0401 | 404.6814 | 397.9308 | 429.7205 | 339.5318 | 361.5719 | 224.0553 | 221.1255 | 164.6186 | 191.5614 | 65.21038 | 24.72029 |
| 42 | 1525.813 | 663.7779 | 654.3341 | 849.0524 | 581.5966 | 626.0333 | 332.0933 | 347.1013 | 243.9437 | 317.6548 | 100.3781 | 52.62382 |

# Fig. 2R

| shControl | shUSP12 |
|---|---|
| 1.031 | 0.314 |
| 0.659 | 0.265 |
| 0.577 | 0.222 |
| 0.541 | 0.184 |
| 0.552 | 0.071 |
| 0.43 | 0.036 |

Fig. 3B

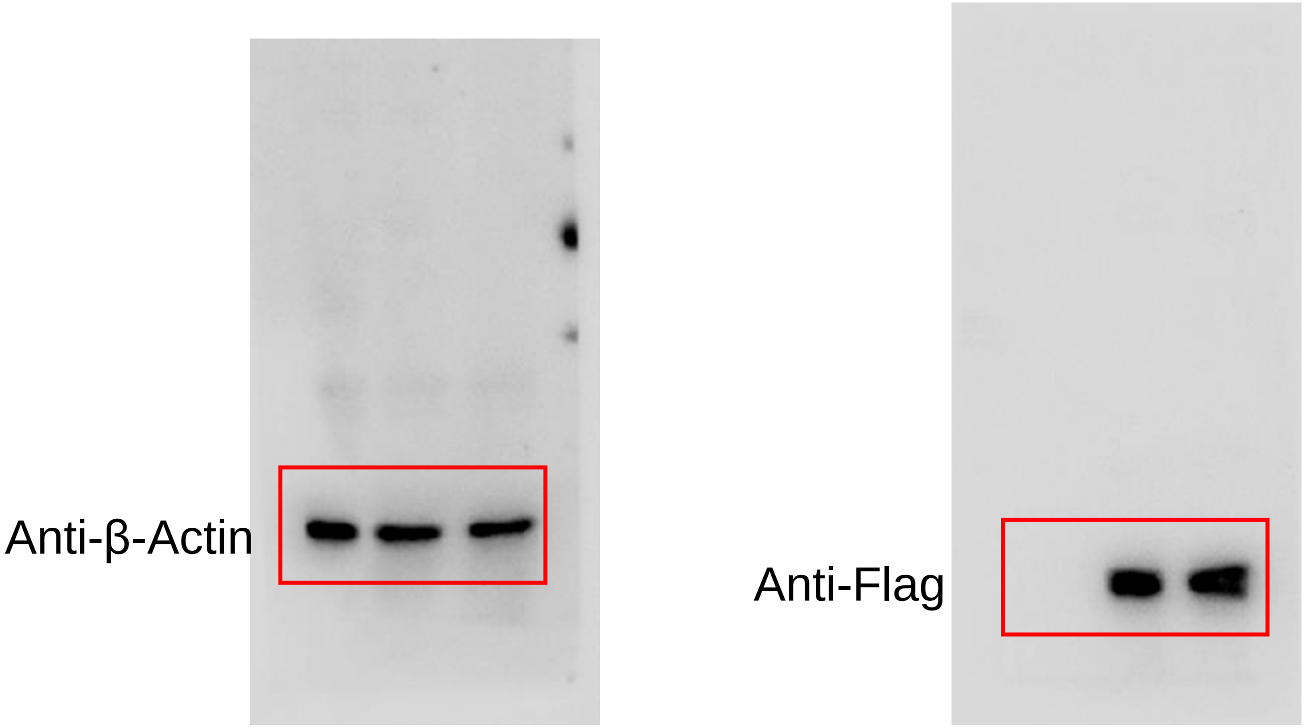

Fig. 3C

| Time (Days) | Flag | | | Flag-USP12$^{WT}$ | | | Flag-USP12$^{C48S}$ | | |
|---|---|---|---|---|---|---|---|---|---|
| 0 | 0.9205 | 0.9886 | 1.0909 | 1.1724 | 0.931 | 0.8966 | 0.7982 | 1.0183 | 1.1835 |
| 1 | 2.1818 | 2.7273 | 3.1364 | 4.3448 | 4.3103 | 4.8276 | 2.8349 | 3.3028 | 2.8349 |
| 2 | 4.2955 | 5.0455 | 5.9659 | 7.7586 | 7.6552 | 8.2414 | 4.6239 | 5.8349 | 6.055 |
| 3 | 8.1818 | 7.3636 | 7.7727 | 11.655 | 13.483 | 12.207 | 7.3761 | 8.367 | 8.8349 |

Fig. 3E

| Flag | Flag-USP12$^{WT}$ | Flag-USP12$^{C48S}$ |
|---|---|---|
| 235 | 480 | 250 |
| 244 | 462 | 241 |
| 238 | 474 | 236 |

| Flag | Flag-USP12$^{WT}$ | Flag-USP12$^{C48S}$ |
|---|---|---|
| 280 | 440 | 300 |
| 277 | 467 | 314 |
| 305 | 424 | 289 |

Fig. 3G

| | Flag | | | Flag-USP12$^{WT}$ | | | Flag-USP12$^{C48S}$ | | |
|---|---|---|---|---|---|---|---|---|---|
| G0/G1 | 69.46 | 67.22 | 69.37 | 60.66 | 57.16 | 58.22 | 68.1 | 69.17 | 67.64 |
| S | 22.34 | 21.09 | 21.44 | 25.68 | 26.34 | 25.91 | 23.09 | 25.2 | 24.08 |
| G2/M | 8.2 | 11.69 | 9.19 | 13.66 | 16.5 | 15.87 | 8.81 | 5.63 | 8.28 |

Fig. 3I

| Flag | Flag-USP12$^{WT}$ | Flag-USP12$^{C48S}$ |
|---|---|---|
| 53.5 | 39.53 | 51.6 |
| 50.74 | 37.15 | 50.44 |
| 52.41 | 36.22 | 54.17 |

Fig. 4A AGS

Anti-β-Actin

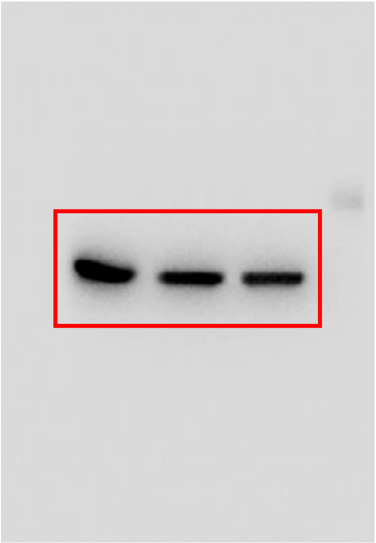

Anti-YAP

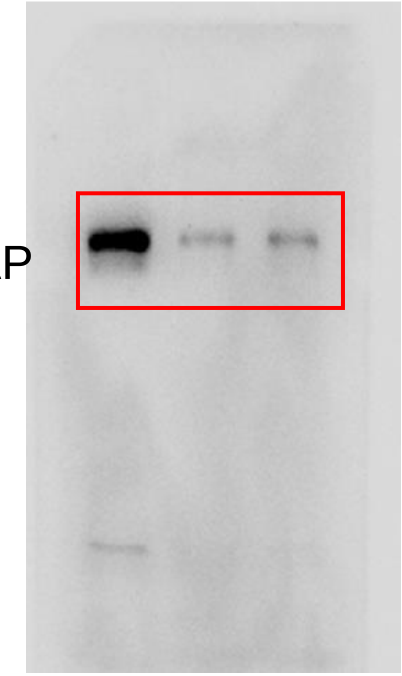

Anti-USP12

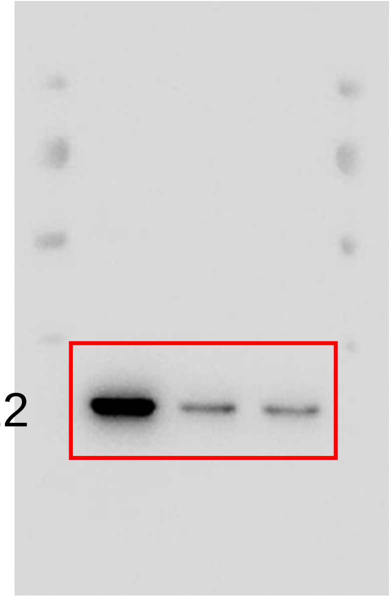

Fig. 4A MKN74

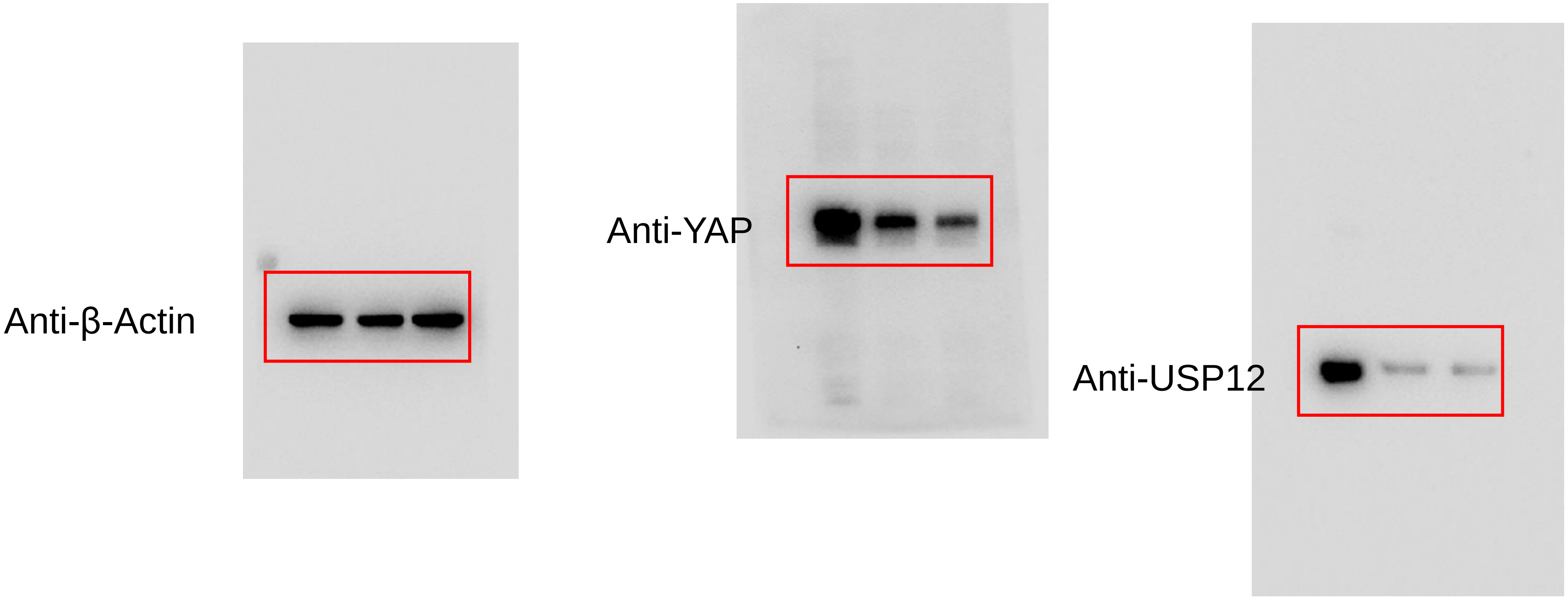

Fig. 4B

| | siControl | | | siUSP12#1 | | | siUSP12#2 | | |
|---|---|---|---|---|---|---|---|---|---|
| CTGF | 1.049068 | 0.995377 | 0.955555 | 0.30654 | 0.376801 | 0.334116 | 0.425075 | 0.430474 | 0.322954 |
| CYR61 | 1.13762 | 0.964274 | 0.898105 | 0.40197 | 0.425438 | 0.446128 | 0.328506 | 0.436815 | 0.342655 |

Fig. 4C

| | siControl | | | siUSP12#1 | | | siUSP12#2 | | |
|---|---|---|---|---|---|---|---|---|---|
| CTGF | 1.035308 | 1.047928 | 0.916764 | 0.314543 | 0.273541 | 0.327951 | 0.359895 | 0.338685 | 0.282732 |
| CYR61 | 0.92851 | 1.168531 | 0.902959 | 0.302625 | 0.310317 | 0.331462 | 0.315169 | 0.342395 | 0.302772 |

# Fig. 4D

| siControl | siUSP12#1 | siUSP12#2 |
|---|---|---|
| 1.102 | 0.301 | 0.312 |
| 0.987 | 0.298 | 0.308 |
| 0.911 | 0.276 | 0.297 |

# Fig. 4E

| siControl | siUSP12#1 | siUSP12#2 |
|---|---|---|
| 1.014 | 0.287 | 0.295 |
| 0.989 | 0.335 | 0.314 |
| 0.997 | 0.304 | 0.338 |

Fig. 4F

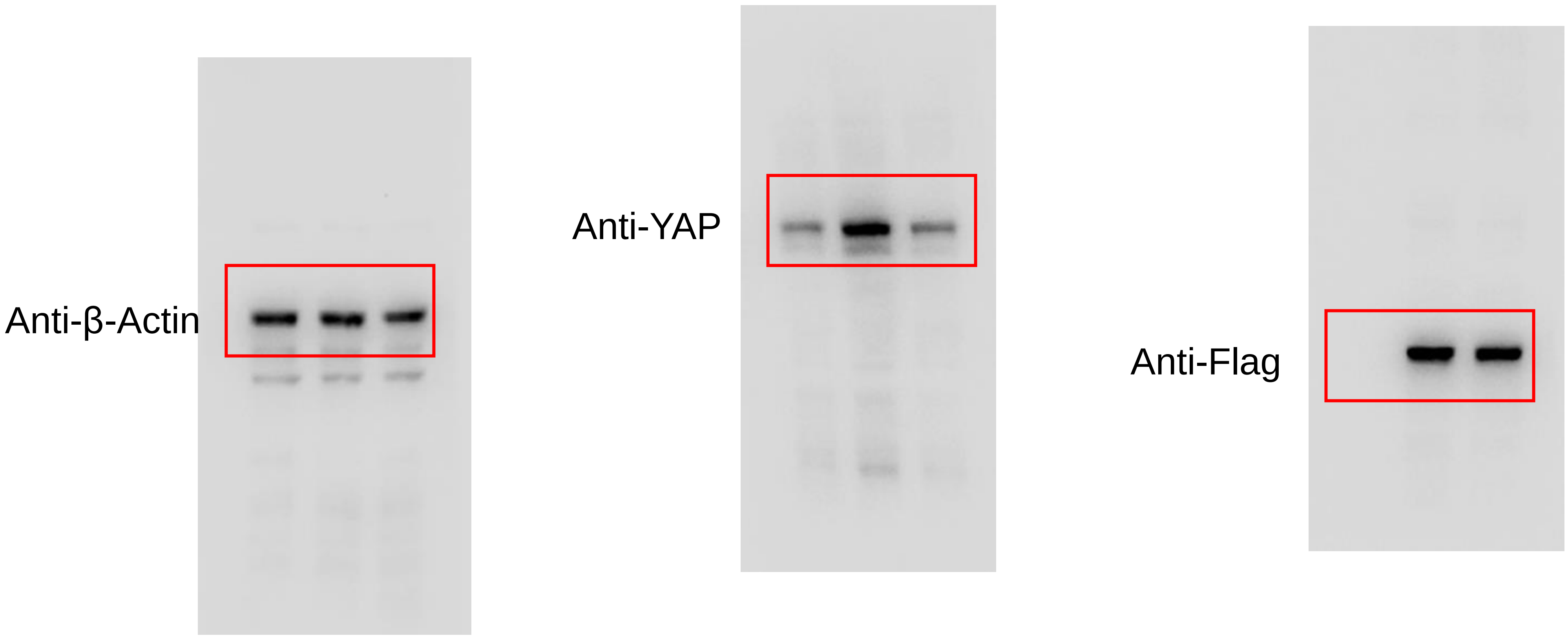

Fig. 4G

| | Flag | | | Flag-USP12$^{WT}$ | | | Flag-USP12$^{C48S}$ | | |
|---|---|---|---|---|---|---|---|---|---|
| CTGF | 1.053891 | 0.898689 | 1.047421 | 3.544299 | 3.207951 | 3.125986 | 1.156272 | 1.339301 | 1.236687 |
| CYR61 | 0.990089 | 0.997824 | 1.012087 | 3.4605 | 3.372802 | 3.175861 | 1.237643 | 1.046517 | 1.168449 |

Fig. 4H

| Flag | Flag-USP12$^{WT}$ | Flag-USP12$^{C48S}$ |
|---|---|---|
| 1.053 | 3.305 | 1.238 |
| 1.125 | 3.247 | 1.067 |
| 0.822 | 3.168 | 1.322 |

Fig. 5A

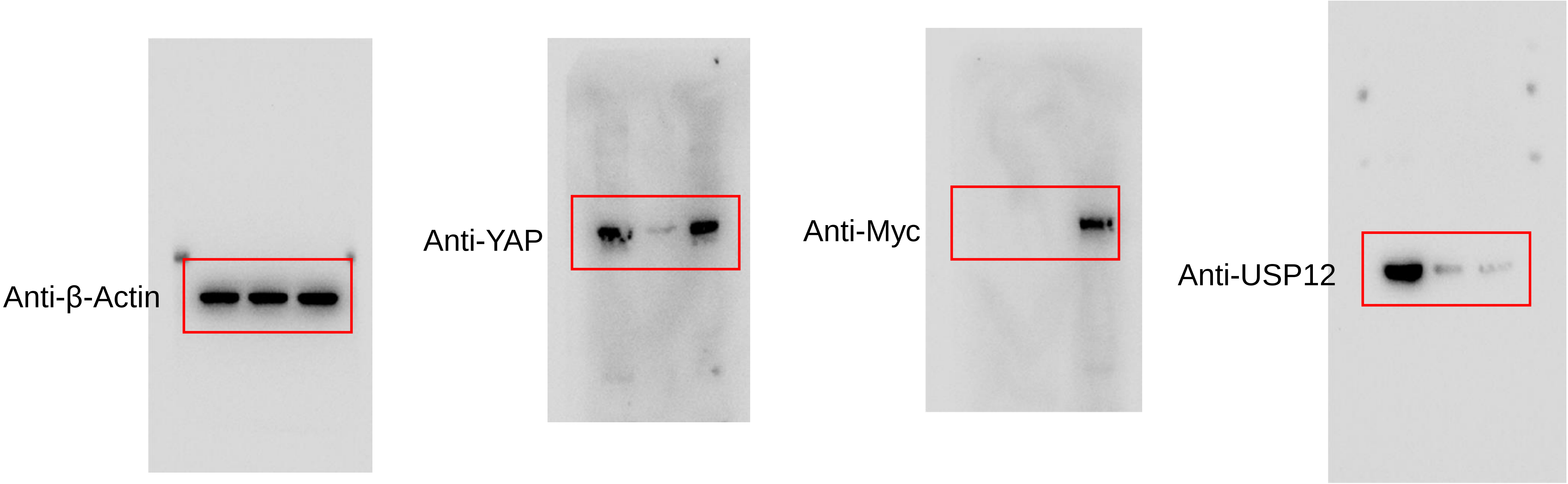

Fig. 5B

| | siControl | | | siUSP12 | | | siUSP12+YAP | | |
|---|---|---|---|---|---|---|---|---|---|
| CTGF | 1.093666 | 1.019159 | 0.887175 | 0.256651 | 0.259405 | 0.314029 | 1.074757 | 1.207619 | 1.119482 |
| CYR61 | 1.031774 | 0.972207 | 0.996019 | 0.254694 | 0.285506 | 0.292179 | 1.109127 | 1.091565 | 1.279183 |

# Fig. 5C

| siControl | siUSP12 | siUSP12+YAP |
|---|---|---|
| 0.992 | 0.257 | 1.135 |
| 0.975 | 0.249 | 1.187 |
| 1.033 | 0.265 | 1.085 |

Fig. 5D

| Time (Days) | siControl | | | siUSP12 | | | siUSP12+YAP | | |
|---|---|---|---|---|---|---|---|---|---|
| 0 | 1.0112 | 1.1798 | 0.809 | 0.8952 | 1.0161 | 1.0887 | 0.866 | 1.2062 | 0.9278 |
| 1 | 4.1124 | 3.3371 | 3.0674 | 2.1532 | 1.7903 | 1.9597 | 3.3402 | 3.2165 | 3.5876 |
| 2 | 6 | 5.4607 | 6.6404 | 2.8548 | 3.121 | 3.4113 | 4.1753 | 5.2887 | 5.9072 |
| 3 | 10.854 | 9.9101 | 9.4719 | 5.4919 | 4.4274 | 5.371 | 9.4021 | 8.3505 | 8.567 |

Fig. 5F

| siControl | siUSP12 | siUSP12+YAP |
|---|---|---|
| 294 | 148 | 235 |
| 277 | 126 | 241 |
| 274 | 120 | 248 |

| siControl | siUSP12 | siUSP12+YAP |
|---|---|---|
| 500 | 220 | 360 |
| 487 | 208 | 401 |
| 466 | 197 | 377 |

Fig. 5H

| | siControl | | | siUSP12 | | | siUSP12+YAP | | |
|---|---|---|---|---|---|---|---|---|---|
| G0/G1 | 61.99 | 60.04 | 58.87 | 70.43 | 69.88 | 74.07 | 63.48 | 63.11 | 61.24 |
| S | 26.88 | 25.41 | 27.06 | 23.51 | 24.1 | 21.47 | 25.8 | 26.14 | 24.41 |
| G2/M | 11.13 | 14.55 | 14.07 | 6.07 | 6.02 | 4.46 | 10.72 | 10.75 | 14.35 |

Fig. 5J

| siControl | siUSP12 | siUSP12+YAP |
|---|---|---|
| 26.44 | 41.5 | 24.6 |
| 25.12 | 40.1 | 25.55 |
| 24.33 | 43.08 | 23.04 |

# Fig. 5L

| Time (Days) | shControl | | | | | | shUSP12 | | | | | | shUSP12+YAP | | | | | |
|---|---|---|---|---|---|---|---|---|---|---|---|---|---|---|---|---|---|---|
| 0 | 0 | 0 | 0 | 0 | 0 | 0 | 0 | 0 | 0 | 0 | 0 | 0 | 0 | 0 | 0 | 0 | 0 | 0 |
| 14 | 80.19692 | 73.94181 | 67.32605 | 35.73832 | 32.177 | 11.70332 | 8.350926 | 6.099377 | 6.286382 | 4.957686 | 6.633288 | 4.262306 | 26.82671 | 18.45332 | 10.42841 | 10.54798 | 10.41152 | 9.962032 |
| 21 | 144.3287 | 132.646 | 110.5882 | 72.09938 | 71.5529 | 37.34417 | 16.49833 | 12.89406 | 16.74416 | 7.720313 | 11.64964 | 6.39744 | 57.60206 | 38.23558 | 20.44655 | 20.26924 | 22.28537 | 24.20532 |
| 28 | 267.7005 | 229.9791 | 189.9513 | 116.3139 | 109.4627 | 62.02182 | 34.80358 | 27.95666 | 34.82566 | 17.0092 | 23.23575 | 13.33275 | 99.87573 | 65.6215 | 38.86934 | 36.98834 | 40.09486 | 53.4224 |
| 35 | 421.8389 | 356.7772 | 314.099 | 177.7745 | 172.2381 | 98.96486 | 63.94032 | 48.93868 | 58.01142 | 28.39601 | 38.67712 | 25.26801 | 164.3388 | 119.3844 | 73.375 | 76.11084 | 75.58824 | 86.15611 |
| 42 | 571.0273 | 538.4672 | 527.3281 | 284.2271 | 280.645 | 153.3645 | 99.90747 | 80.73413 | 86.022 | 72.69773 | 69.26258 | 40.89904 | 263.5091 | 211.9201 | 113.0824 | 131.0076 | 120.9245 | 131.1663 |

# Fig. 5M

| shControl | shUSP12 | shUSP12+YAP |
|---|---|---|
| 0.475 | 0.067 | 0.252 |
| 0.377 | 0.055 | 0.171 |
| 0.289 | 0.053 | 0.111 |
| 0.27 | 0.049 | 0.094 |
| 0.183 | 0.044 | 0.095 |
| 0.137 | 0.031 | 0.105 |

Fig. 6B

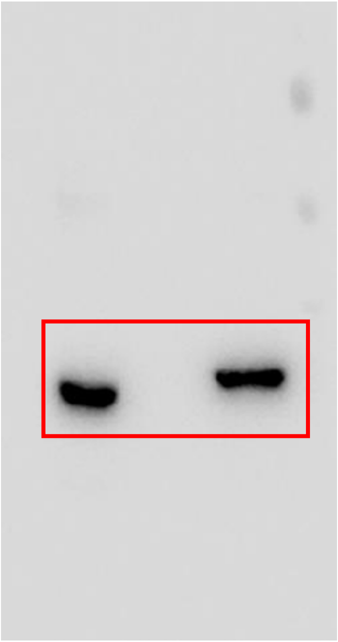


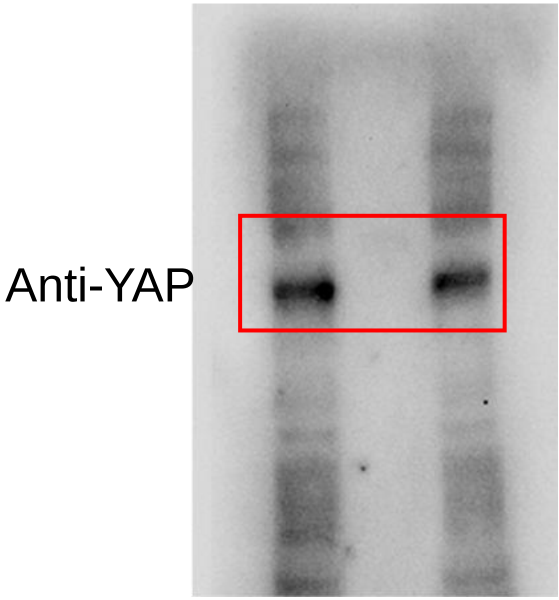

Fig. 6C

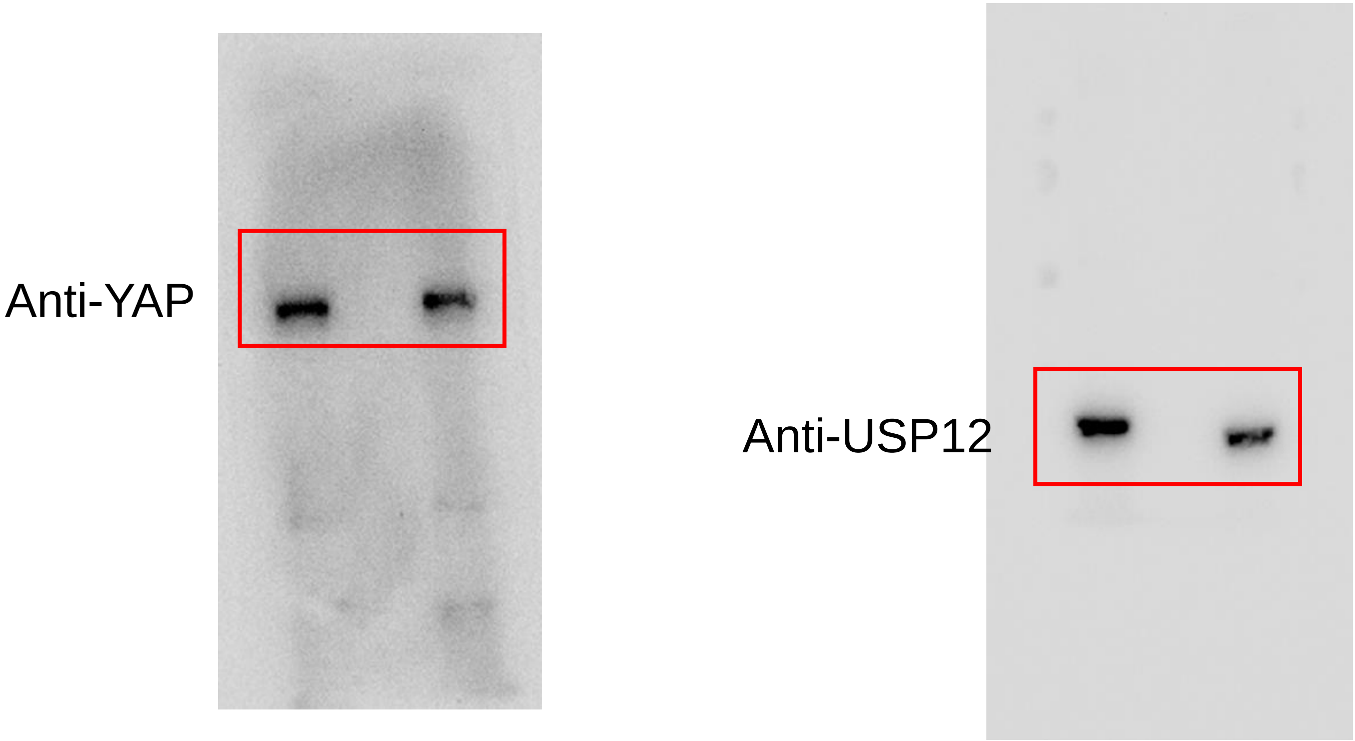

Fig. 6D

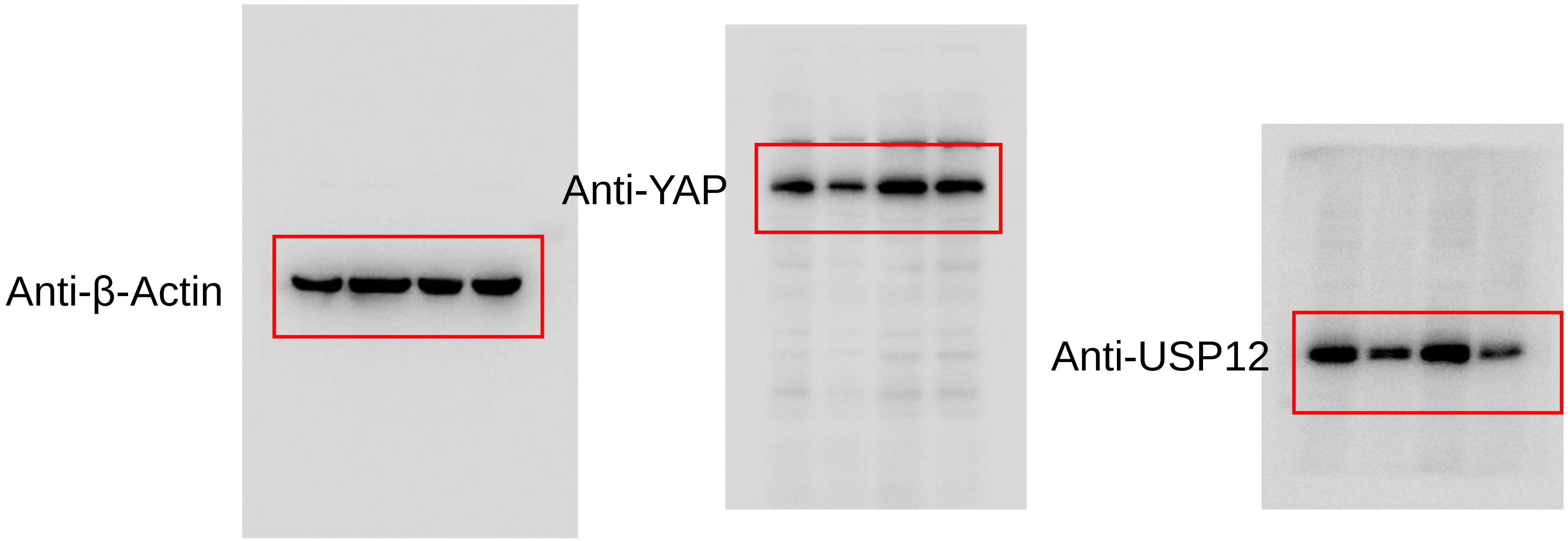

Fig. 6E

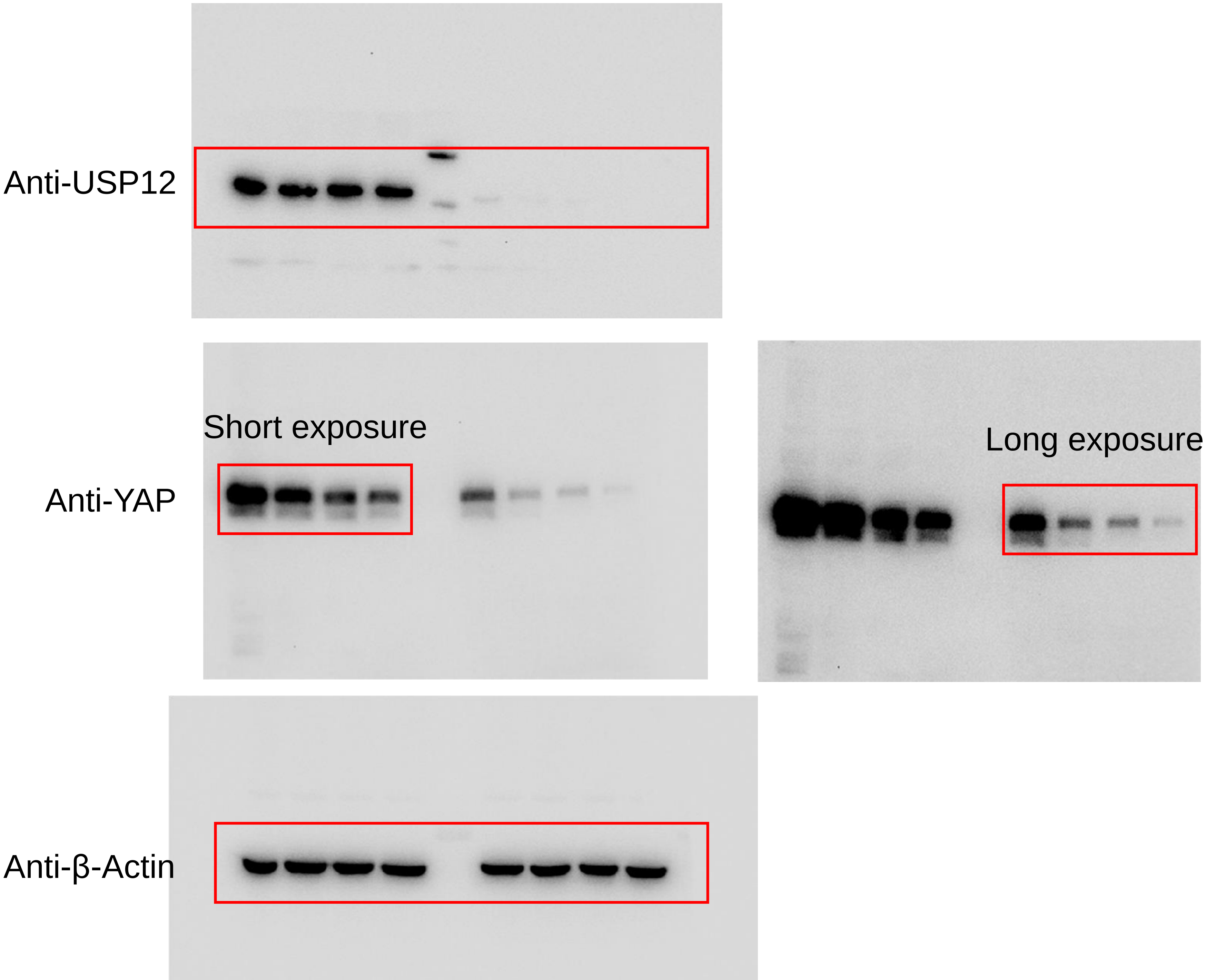
Anti-USP12
Anti-YAP
Short exposure
Long exposure
Anti-β-Actin

Fig. 6G

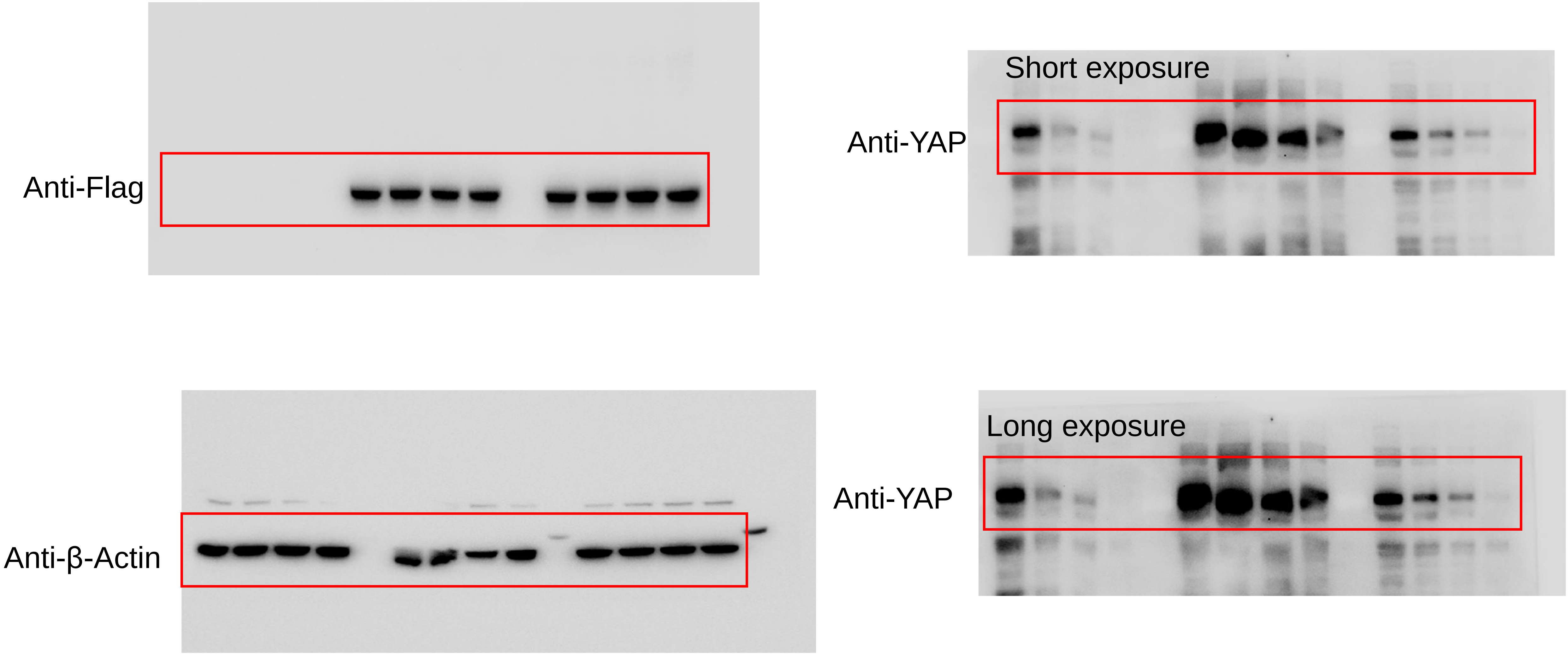
Anti-Flag
Short exposure
Anti-YAP
Anti-β-Actin
Long exposure
Anti-YAP

Fig. 7A

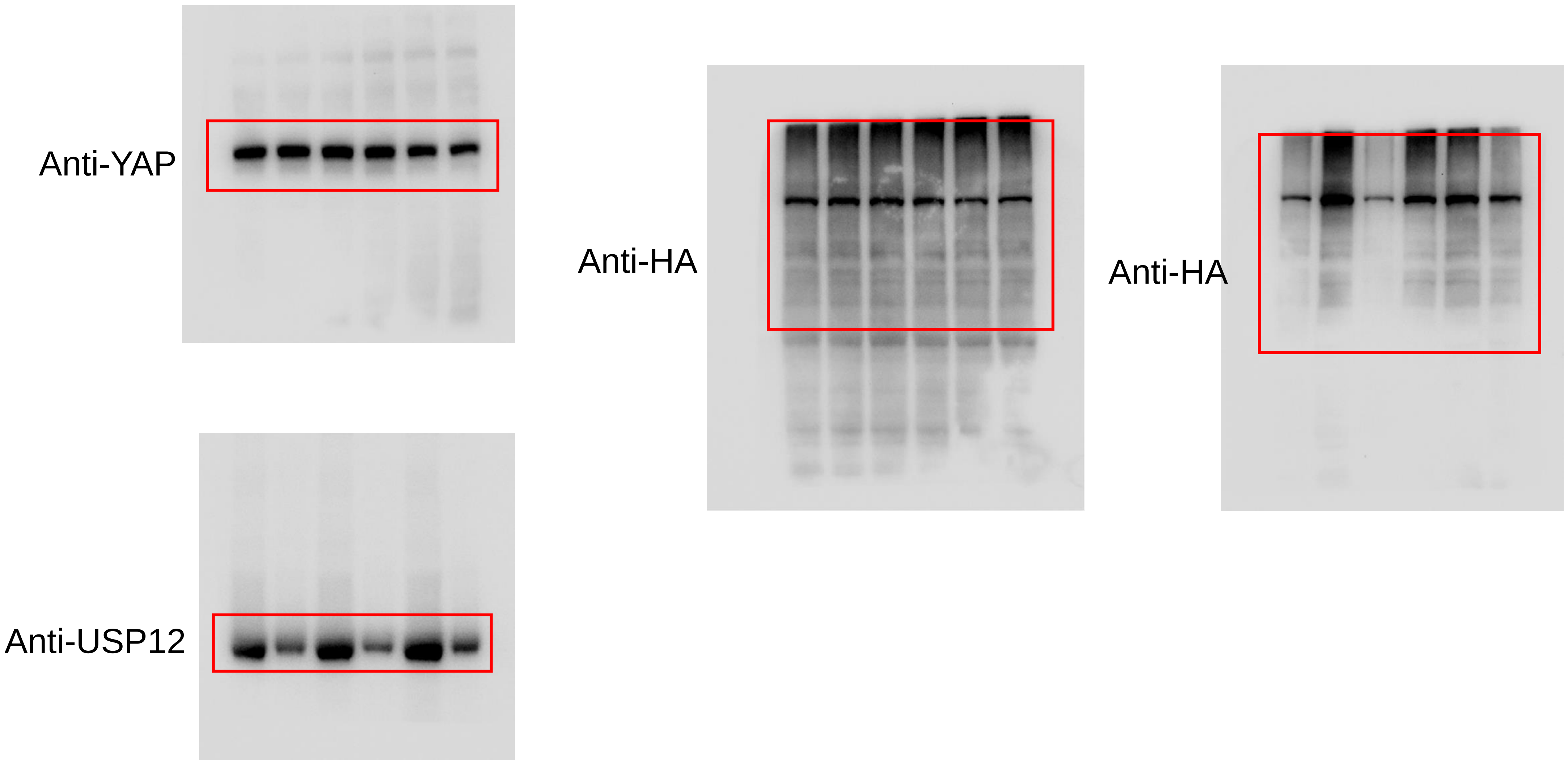

Fig. 7B

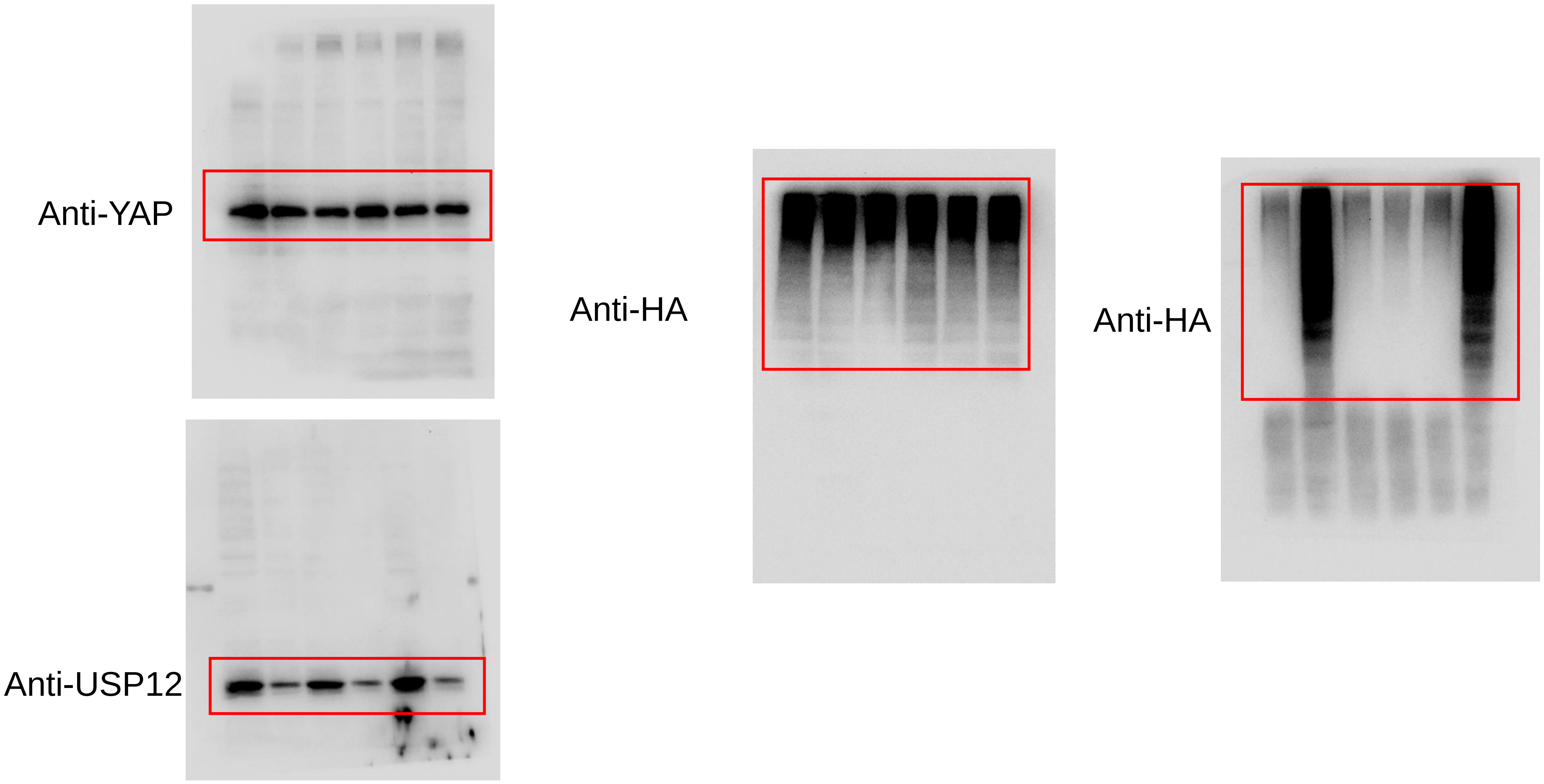

Fig. 7C

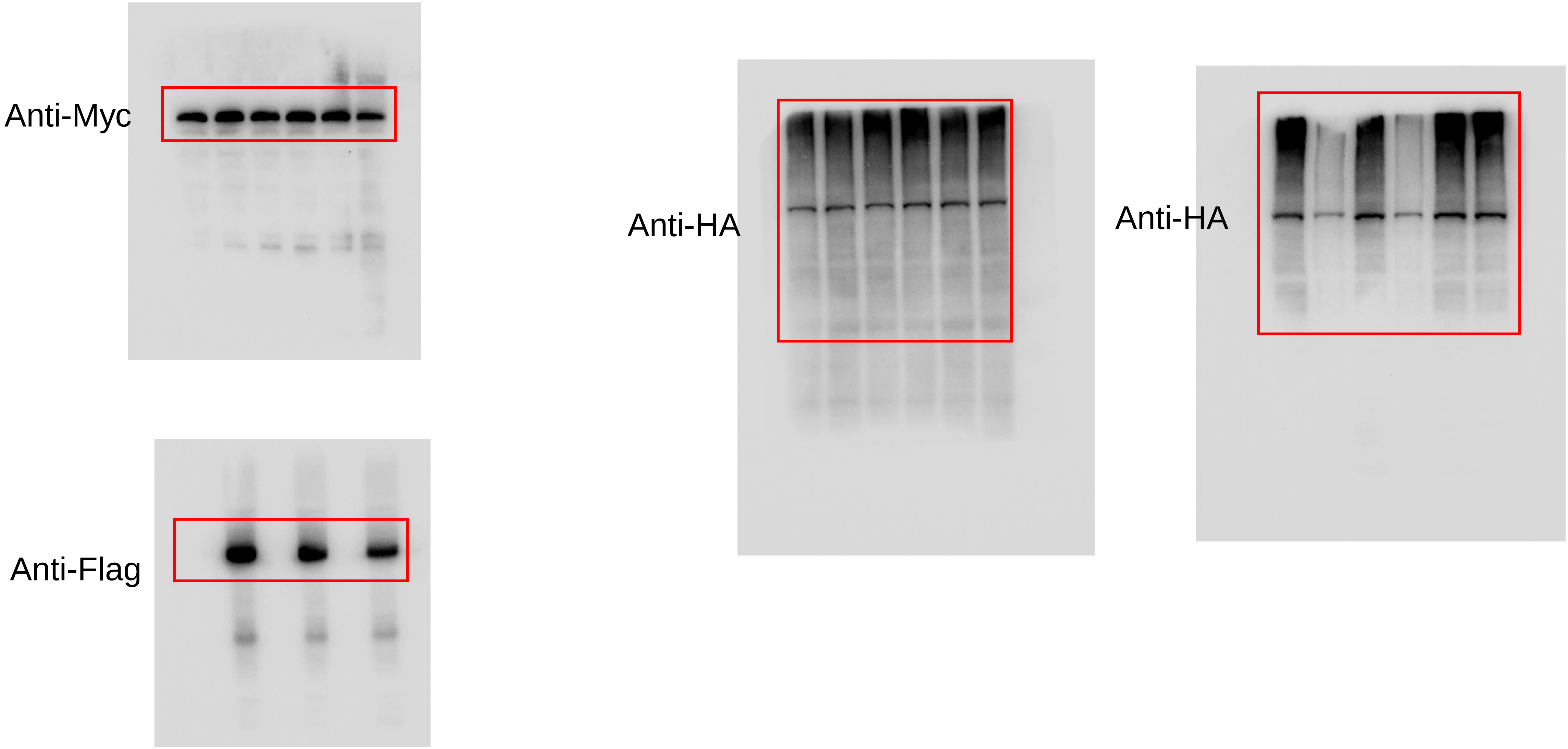

Fig. 7D

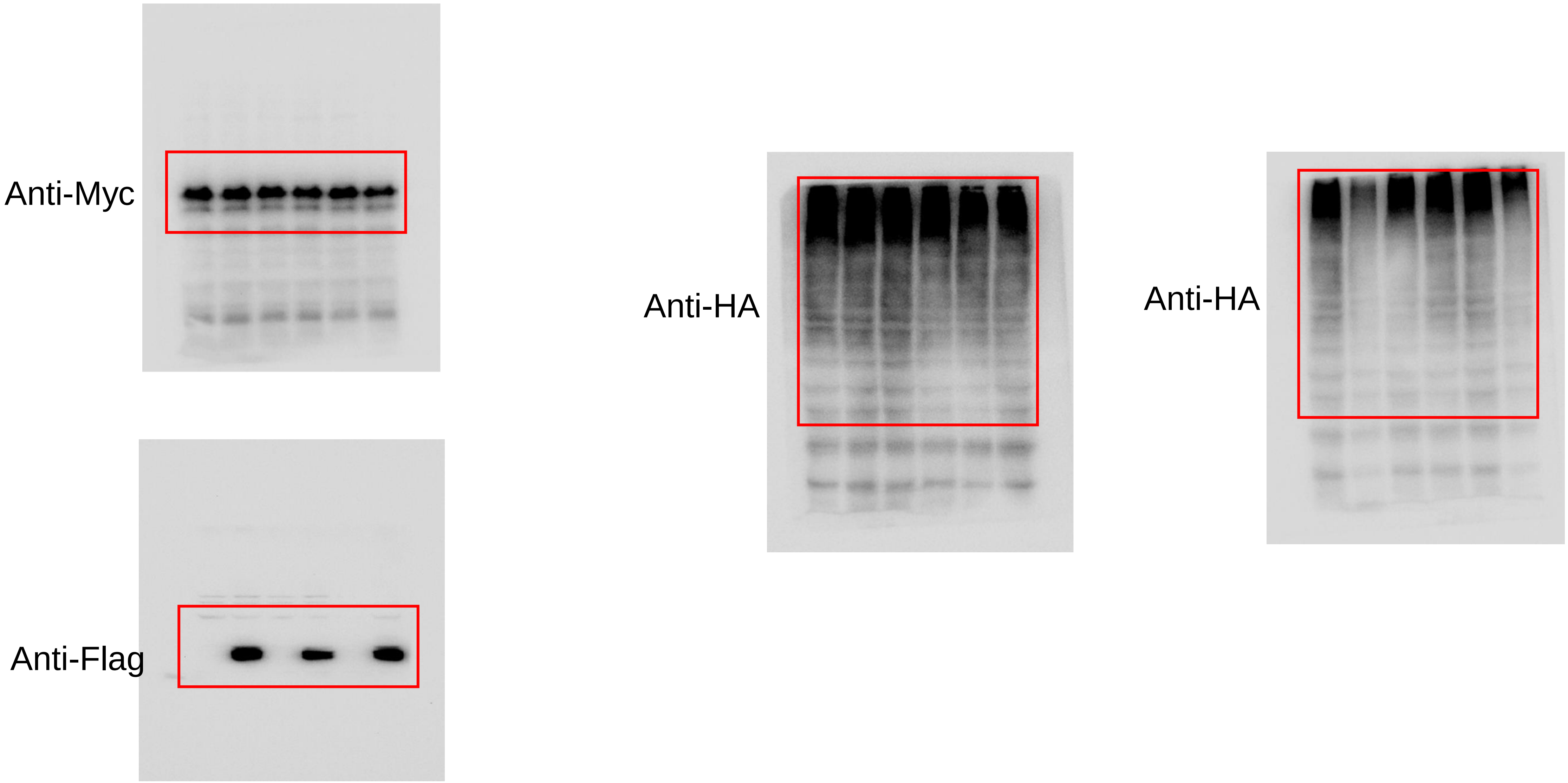

Fig. 7E

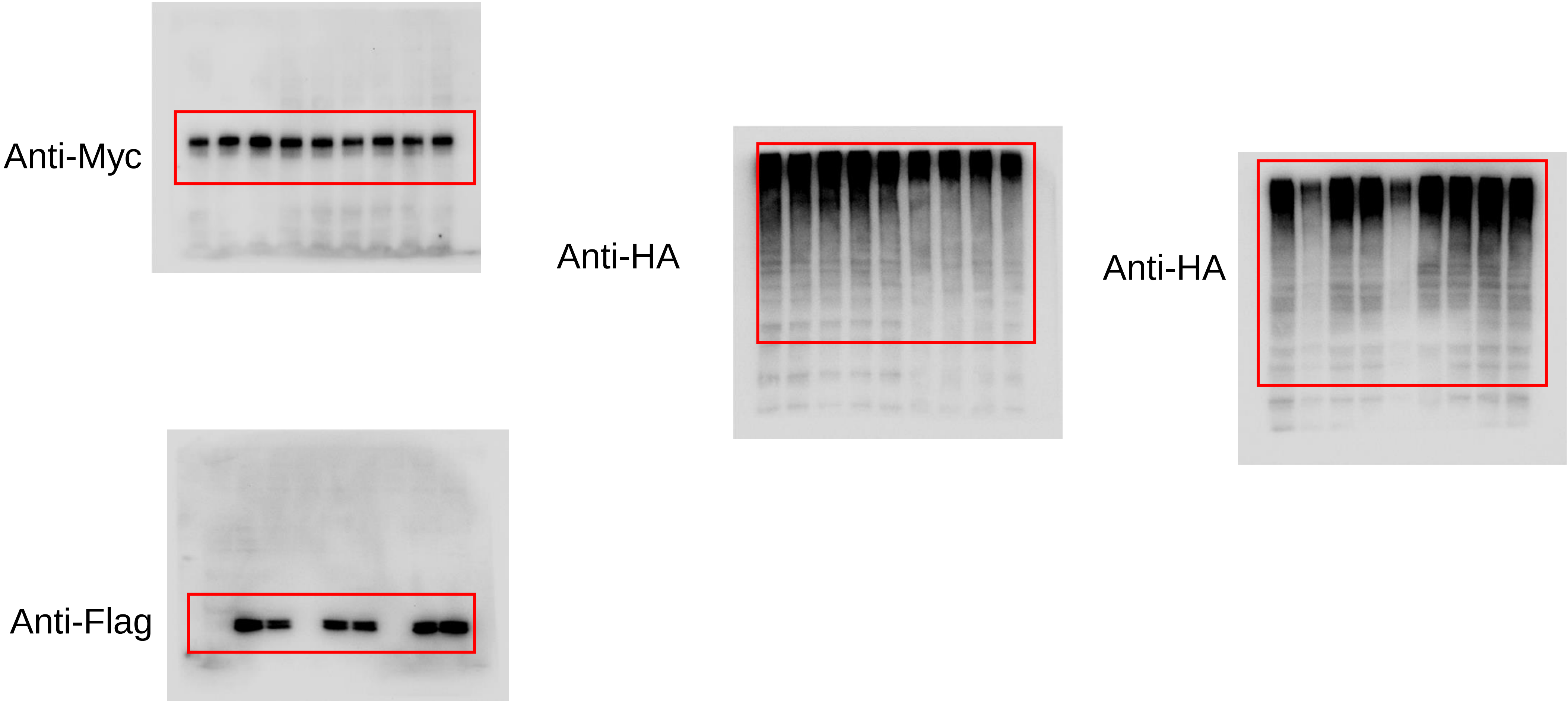

Fig. 7F

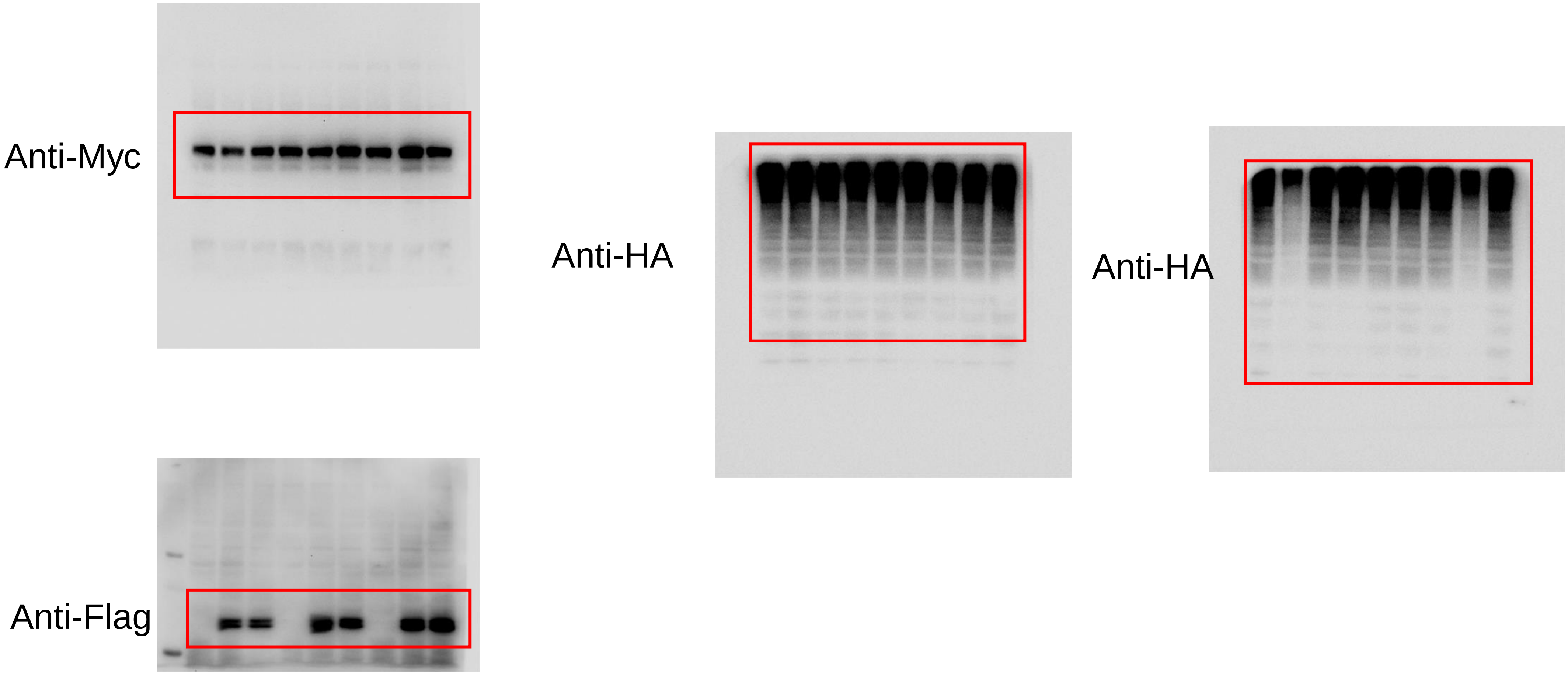

Fig. 7G

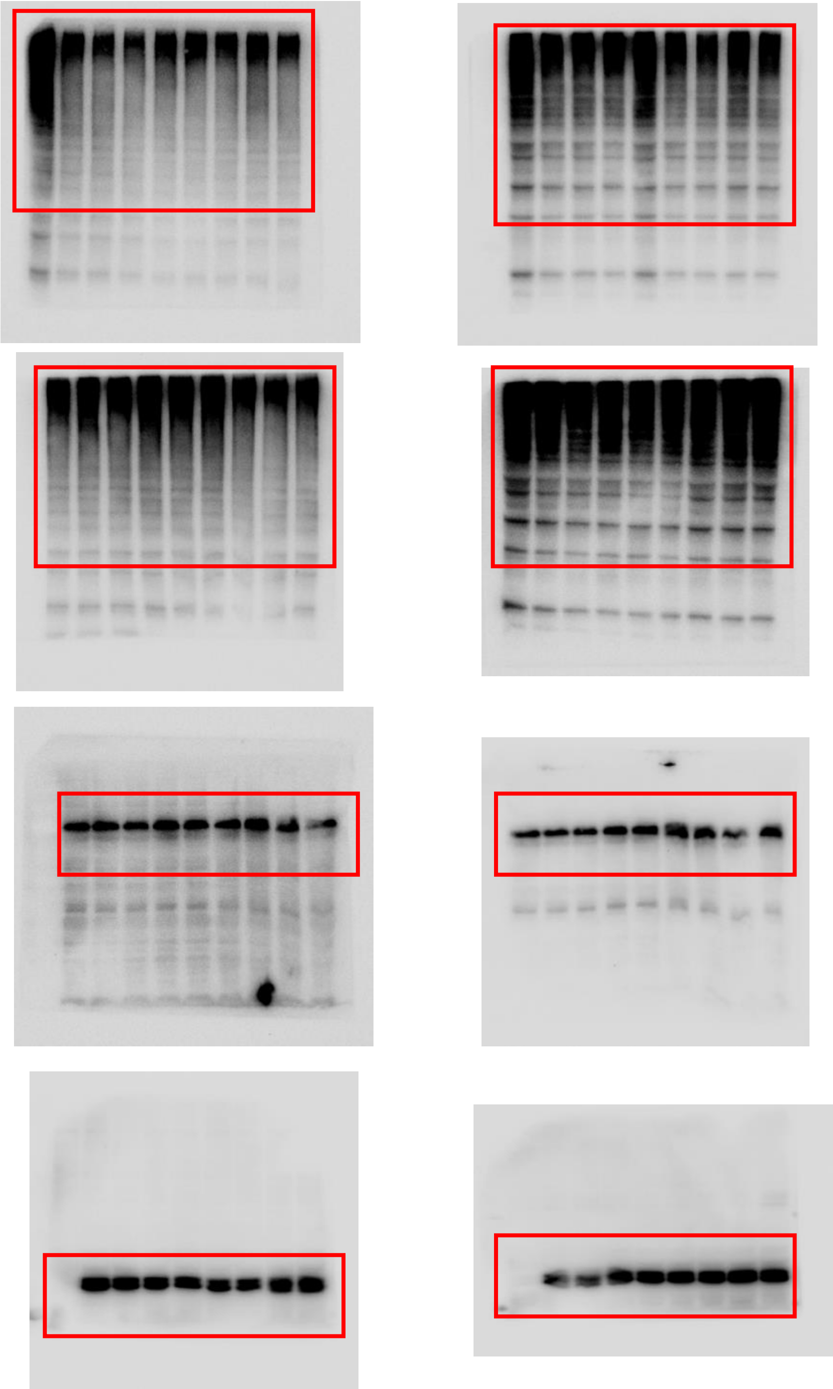

Anti-HA

Anti-HA

Anti-Myc

Anti-Flag

Fig. S1A

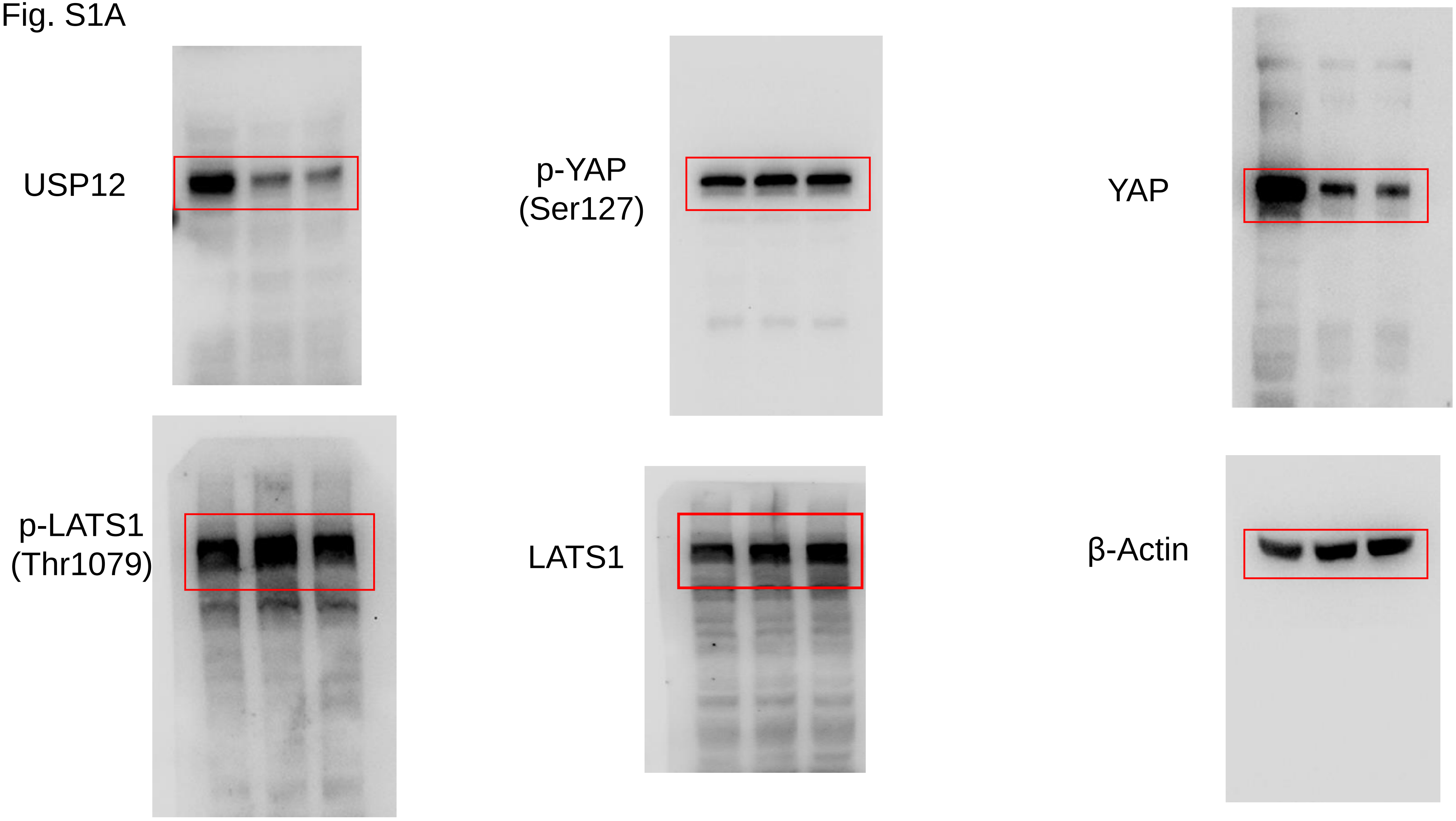

Fig. S3A

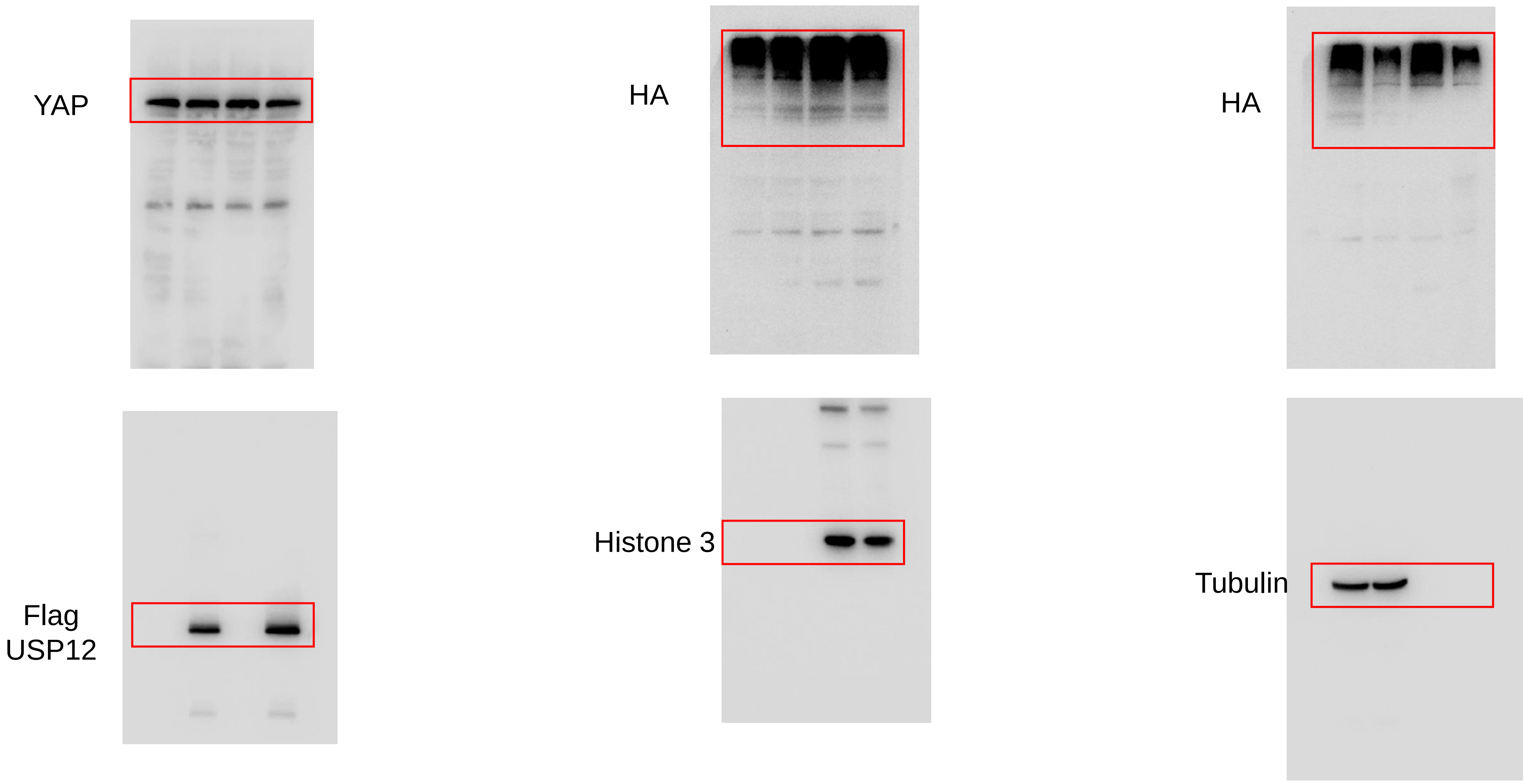

Supplement: Supplementary file 5 — Original Data File [file 41420_2024_1943_MOESM5_ESM.pdf]
